# Thermal biology of mosquito‐borne disease

**DOI:** 10.1111/ele.13335

**Published:** 2019-07-08

**Authors:** Erin A. Mordecai, Jamie M. Caldwell, Marissa K. Grossman, Catherine A. Lippi, Leah R. Johnson, Marco Neira, Jason R. Rohr, Sadie J. Ryan, Van Savage, Marta S. Shocket, Rachel Sippy, Anna M. Stewart Ibarra, Matthew B. Thomas, Oswaldo Villena

**Affiliations:** ^1^ Department of Biology Stanford University 371 Serra Mall Stanford CA USA; ^2^ Department of Entomology and Center for Infectious Disease Dynamics Penn State University University Park PA 16802 USA; ^3^ Department of Geography and Emerging Pathogens Institute University of Florida Gainesville FL USA; ^4^ Department of Statistics Virginia Polytechnic and State University 250 Drillfield Drive Blacksburg VA USA; ^5^ Center for Research on Health in Latin America (CISeAL) Pontificia Universidad Católica del Ecuador Quito Ecuador; ^6^ Department of Biological Sciences Eck Institute of Global Health Environmental Change Initiative University of Notre Dame, Notre Dame IN USA; ^7^ School of Life Sciences University of KwaZulu‐Natal Durban South Africa; ^8^ Department of Ecology and Evolutionary Biology and Department of Biomathematics University of California Los Angeles Los Angeles CA 90095 USA; ^9^ Santa Fe Institute 1399 Hyde Park Rd Santa Fe NM 87501 USA; ^10^ Institute for Global Health and Translational Sciences SUNY Upstate Medical University Syracuse NY13210 USA

**Keywords:** Arbovirus, climate change, dengue virus, malaria, mosquito, Ross River virus, temperature, thermal performance curve, West Nile virus, Zika virus

## Abstract

Mosquito‐borne diseases cause a major burden of disease worldwide. The vital rates of these ectothermic vectors and parasites respond strongly and nonlinearly to temperature and therefore to climate change. Here, we review how trait‐based approaches can synthesise and mechanistically predict the temperature dependence of transmission across vectors, pathogens, and environments. We present 11 pathogens transmitted by 15 different mosquito species – including globally important diseases like malaria, dengue, and Zika – synthesised from previously published studies. Transmission varied strongly and unimodally with temperature, peaking at 23–29ºC and declining to zero below 9–23ºC and above 32–38ºC. Different traits restricted transmission at low versus high temperatures, and temperature effects on transmission varied by both mosquito and parasite species. Temperate pathogens exhibit broader thermal ranges and cooler thermal minima and optima than tropical pathogens. Among tropical pathogens, malaria and Ross River virus had lower thermal optima (25–26ºC) while dengue and Zika viruses had the highest (29ºC) thermal optima. We expect warming to increase transmission below thermal optima but decrease transmission above optima. Key directions for future work include linking mechanistic models to field transmission, combining temperature effects with control measures, incorporating trait variation and temperature variation, and investigating climate adaptation and migration.

## Introduction

Pathogens transmitted by biting arthropods – vector‐borne diseases – comprise a major burden of human, animal, and plant diseases worldwide. Transmission of these pathogens is tightly linked to the ecology of vector populations, including biting behaviour, competence for transmitting the pathogen, survival, and life history. This vector ecology depends in part on climate, habitat, and host density (Gatton *et al.* 2005; Bi *et al.* 2009; Paaijmans *et al.* 2010b, 2011; Werner *et al.* 2012; Mordecai *et al.* 2013; Stewart Ibarra *et al.* 2013; Mordecai *et al.* 2017; Paull *et al.* 2017; Shapiro *et al.* 2017; Thomson *et al.* 2017; Shocket *et al.* 2018; Tesla *et al.* 2018). Since the turn of the 20th century, when scientists and physicians discovered that mosquitoes transmit malaria and yellow fever, biologists have recognised that temperature drives vector‐borne disease transmission. Ambient temperatures alter mosquito lifespan and the period after ingesting an infectious blood meal before a mosquito becomes infectious (the extrinsic incubation period); these traits in turn affect the rate of pathogen transmission (Cox 2010; Smith *et al.* 2012). Medical entomologists have since characterised how temperature also affects the rates of biting, reproduction, development, and survival across vector life stages, and the probability of becoming infectious after biting an infectious host (i.e., vector competence) (Thomas & Blanford 2003; Shapiro *et al.* 2017). Temperature shapes transmission via its effects on all of these traits, promoting transmission at intermediate optimal temperatures and suppressing it beyond lower and upper thermal limits (Craig *et al.* 1999; Lafferty 2009).

Although temperature affects the transmission of all arthropod‐borne pathogens, here we focus on mosquito‐borne diseases because they pose a major worldwide health burden and because the effects of temperature are perhaps best recognised in these pathogens, particularly in light of climate change (Martens *et al.* 1997; Craig *et al.* 1999; Pascual *et al.* 2006; Pascual & Bouma 2009; Parham & Michael 2010; Alonso *et al.* 2011; Rohr *et al.* 2011; Stewart Ibarra *et al.* 2013; Siraj *et al.* 2014; Ryan *et al.* 2019). Several important gaps limit our ability to understand the effect of temperature on mosquito‐borne disease (Parham *et al.* 2015). In this paper, we summarise scientific knowledge about the role of temperature in mosquito‐borne disease transmission, identify critical gaps, and chart a course for future research in the context of changing climate and emerging diseases. First, we outline fundamental concepts in vector thermal biology. Then, we illustrate how these concepts can be applied by synthesising recent trait‐based research on the effects of temperature on multiple mosquito‐borne parasites and viruses and by making quantitative comparisons. Finally, we discuss implications and predictions for transmission under climate change, open questions to shape future research on the thermal biology of mosquito‐borne disease (Box 1), and extensions to other types of arthropod vectors. We aim to identify generalities in the effects of temperature on mosquito‐borne disease transmission, leaving more system‐specific processes such as precipitation, immature vector habitat, host distribution and behaviour, immune dynamics, socioeconomic factors, and vector control for future work.

Box 1 **Key open questions to guide the future of vector‐borne disease thermal biology research.**

Building on over a century of progress on climate‐driven vector‐borne disease research, several important questions remain to guide the next century of research.

- Across species and populations, what tradeoffs constrain the evolution and acclimation of thermal performance curves?
- Is variation in thermal performance curves greatest in the magnitude, optimum, breadth, or limits?
- How does variation in climate extremes interact with changes in climate means and variances to affect trait performance and transmission?
- What is the potential for thermal performance to adapt to warming temperatures, and which aspects of thermal performance have the most adaptive potential: thermal optima, performance breadth, or heat or cold tolerance?
- What physiological, genetic, and environmental pathways drive variation in thermal performance among individuals, populations, species, and traits, and how predictable is this variation?
- How accurately can thermal performance curves that are derived from constant temperature experiments be integrated across temperature variation to predict performance under varying temperatures?
- How can trait‐based model predictions best be combined with observed dynamics of human cases to infer and predict the role of temperature in disease dynamics?
- At what geographic and temporal scales is temperature most useful as a predictor for vector‐borne disease dynamics? What drivers interact with temperature to drive disease dynamics?
- Are warming‐driven declines in transmission in formerly optimal ranges already occurring? How do we isolate putative temperature‐driven declines from the impacts of public health interventions, rainfall, humidity, land use, human immunity, and human behaviour?
- How well matched are species’ thermal response curves to their environments and how well are thermal responses of vectors and parasites matched to each other? How important is this matching or mismatching?

### Foundational concepts in thermal biology

Temperature limits the geographic range and magnitude of mosquito‐borne disease transmission via its effects on mosquito and pathogen traits (Martens *et al.* 1997; Craig *et al.* 1999; Thomas & Blanford 2003; Parham & Michael 2010; Mordecai *et al.* 2013, 2017; Paull *et al.* 2017; Shapiro *et al.* 2017; Shocket *et al.* 2018; Tesla *et al.* 2018). Transmission cannot occur at temperatures that prohibit mosquito or pathogen survival, development, reproduction, or metabolism. Within the range of permissive temperatures, the nonlinear influence of temperature on mosquito and pathogen traits affects the magnitude of transmission (Thomas & Blanford 2003). Determining the effects of temperature on transmission requires identifying temperatures that optimise the tradeoffs between different temperature‐dependent traits of mosquitoes and pathogens.

**Figure 1:**
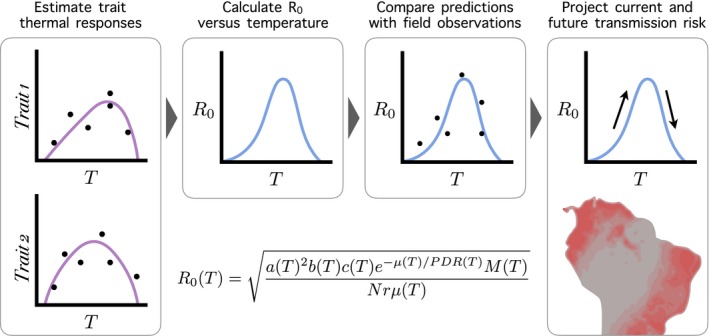
The trait‐based approach to understanding effects of temperature on vector‐borne disease transmission. In this approach, we derive trait thermal performance curves from experimental data, synthesise their combined temperature‐dependent effect on *R_0_*, validate the model with independent field observations, and project predicted temperature suitability for transmission onto current and future climates.

Extensive empirical and theoretical work has established that most physiological and life history traits respond nonlinearly to temperature – increasing from zero at a thermal minimum approximately exponentially up to an optimum before declining back to zero at a thermal maximum (Huey & Berrigan 2001; Angilletta 2009; Dell *et al.* 2011) (Fig. 1). This unimodal or hump‐shaped relationship is nearly universal across measured responses from ectotherm taxa and traits (Dell *et al.* 2011) and is predicted from first principles of enzyme kinetics and physiology (Huey & Berrigan 2001; Angilletta 2009; Kingsolver 2009; Huey & Kingsolver 2011; Amarasekare & Savage 2012). Moreover, the rates of increase and decline in performance with temperature are tightly constrained for many traits. From the metabolic theory of ecology (Brown *et al.* 2004), the approximate exponential rate of increase (the Boltzmann–Arrhenius constant) ranges from 0.6 to 0.7 eV for most metabolically related traits and taxa, while the constant for exponential decline above the optimum is approximately 1.2 eV (Brown *et al.* 2004; Dell *et al.* 2011). Metabolic theory of ecology has predicted host and parasite traits that affect parasite dynamics in microsporidia and helminths (Hechinger 2013; Molnár *et al.* 2013; Kirk *et al.* 2018, 2019; O’Connor & Bernhardt 2018). Whether these canonical values from metabolic theory apply to the traits of mosquitoes and pathogens that drive vector‐borne disease transmission is unknown (Molnár *et al.* 2013, 2017).

Thermal optima for traits like development, survival, and reproduction affect organismal fitness, and vary with the profile of the environmental temperature (Deutsch *et al.* 2008; Kingsolver 2009). As temperature varies over time, organisms experience temperatures below and above their thermal optima, affecting fitness. Such temperature variation may limit species range boundaries (Overgaard *et al.* 2014). The cost of exceeding thermal optima are higher than the costs of undershooting thermal optima when trait thermal performance curves are cold‐skewed (Bernhardt *et al.* 2018). Since daily and seasonal temperature variation is large in temperate regions, many temperate species have evolved thermal performance curves with optima well above the mean environmental temperature (Deutsch *et al.* 2008; Martin & Huey 2008). This ‘thermal safety margin’ buffers individual and population fitness against temperature fluctuations, particularly the disproportionate cost of temperatures exceeding thermal optima. By contrast, many tropical species, that experience less variation in temperature have low thermal safety margins (Deutsch *et al.* 2008). As a result, climate warming is expected to push environmental temperatures above thermal optima for many organisms, particularly in the tropics (Deutsch *et al.* 2008; Kingsolver 2009). The emergent effect of climate change depends on a population’s thermal performance relative to the current environment, thermal stress tolerance, and ability to adapt or migrate, and the speed and magnitude of climate change (Loarie *et al.* 2009; Paaijmans *et al.* 2013).

From theory and empirical studies, we derive four key thermal biology principles for mosquito‐borne disease:

1. Mosquito and pathogen traits related to survival, development, and reproduction are temperature sensitive.
2. These traits generally respond to temperature unimodally, with lower and upper limits and optima potentially adapted to local environments.
3. Climate change will affect many mosquito and pathogen traits that govern mosquito distribution, abundance, and pathogen transmission.
4. Climate change has the potential to increase, decrease, or have minimal effect on transmission depending on organismal thermal responses, the changing climate regime, and the rate of migration or adaptation.

Temperature defines one dimension of the fundamental ecological niche for mosquito‐borne disease transmission – the range of conditions that is required for transmission to be possible – which also includes immature vector habitat and humidity. Within this fundamental niche, the realised ecological niche for transmission additionally depends on host factors including density, movement, behaviour, demography, susceptibility, control strategies, and exposure to mosquito bites (Gething *et al.* 2010; Rodriguez‐Barraquer *et al.* 2011; Paaijmans & Thomas 2011a; Parham *et al.* 2015; Wesolowski *et al.* 2015; Krisher *et al.* 2016; Metcalf *et al.* 2017; Salje *et al.* 2017, 2018; Jaramillo‐Ochoa *et al.* 2019). Mosquito and pathogen physiological responses to temperature determine fundamental transmission potential, but the realised impact of climate change on disease dynamics also depends on these host population processes, socio‐economics, disease control efforts, or other mitigation measures (Gething *et al.* 2010; Paaijmans & Thomas 2011a; Parham *et al.* 2015; Wesolowski *et al.* 2017). Shared physiological mechanisms imply that mosquito thermal biology is likely to apply generally across systems and scales, whereas host population biology may be more responsive to context‐dependent behavioural or technological adaptation. For these reasons, we focus here on how physiological effects of temperature on mosquito and pathogen traits affect transmission.

To illustrate one approach for applying principles of thermal biology to vector‐borne disease transmission, we synthesise the results of previous research that used trait‐based models derived from experimental data to understand how temperature affects mosquito‐borne disease transmission. This is one application – with several strengths and limitations – of thermal biology concepts that apply more generally across research approaches. We also highlight important caveats that correspond to key extensions and directions for future research (Table 1).

**Table 1:** Challenges and future research directions for the temperature‐dependent *R_0_* approach

Challenge	Future research directions
(1) **Data quality and gaps.** Trait thermal response data are often sparse, requiring models to estimate trait thermal responses from related species or limited numbers of data points or temperatures. What is the quality of data that is truly needed?	Use sensitivity and uncertainty analyses on models to identify key data gaps to prioritise for experiments (Johnson *et al. * 2015; Parham *et al. * 2015).
(2) **Generalities across thermal performance curves.** How much do differences across species matter, given the other sources of variation in transmission in the field?	Use sensitivity analyses on models to identify which traits most influence differences in transmission among species. Increase model validation efforts to measure the degree to which predicted species differences are borne out in the field.
(3) **Relative versus absolute thermal performance curves.** Although the relative *R_0_* approach synthesises how the relative shapes of trait thermal performance curves affect transmission, differences in absolute trait magnitudes could also affect temperature‐dependent transmission. How do relative magnitudes of traits like lifespan, biting rate, and extrinsic incubation rate affect transmission?	Measure traits and/or entomological inoculation rate (EIR) across temperatures in the field to determine how, for example, the relative magnitudes of biting rates, extrinsic incubation rates, and lifespan vary across temperature. Model the effects of absolute differences in trait values on transmission rates, across temperatures.
(4) **Interactive versus additive effects of temperature and other factors on traits.** Humidity, food quality, and other factors affect vector traits simultaneously with temperature. Are these effects additive (scaling the magnitude of the trait thermal performance curve) or interactive (changing the shape, limits, or optimum of the thermal performance curve)?	Experimentally vary temperature and other environmental factors in a factorial design and measure vector and parasite traits (e.g. Bayoh 2001; Murdock *et al. * 2014). Use theory based on physiological mechanisms underlying trait performance to generate hypotheses for additive versus interactive effects of temperature and other factors (Kearney *et al. * 2009).
(5) **Translation of thermal performance curves from laboratory to field.** Vector traits can vary substantially between the laboratory and the field, including longevity, biting, and egg‐laying. Are the laboratory‐estimated thermal performance curves proportional to trait thermal responses in the field?	Validate mechanistic models and laboratory trait measurements in the field. Incorporate field‐based trait estimates into mechanistic models to understand their impacts on transmission.
(6) **Trait interactions across life stages.** Larval rearing conditions such as food availability and temperature can affect body size and physiological condition, which affect adult traits such as longevity, biting rate, and vector competence. How does trait covariance across life stages affect transmission?	Use physiological ecology to model mechanistic linkages among traits based on joint dependence on metabolism, body size, and body condition. Empirically estimate trait covariation across life stages, and incorporate joint distributions of trait thermal performance curves into mechanistic models.
(7) **Relative importance of temperature versus other drivers of traits and transmission.** Temperature is one component in a complex network of causality linking environmental conditions to vector traits and disease transmission. What is the relative importance of these drivers, and how does it vary across transmission settings?	Systematically review epidemiological studies that estimate the importance of temperature and other factors (e.g. Stewart‐Ibarra & Lowe 2013) to see how their importance varies across settings, diseases, and scales (Cohen *et al. * 2016). Use species distribution models (SDMs) to estimate the relative importance of different factors for explaining current distributions of vectors and diseases. Use mechanistic and statistical models to estimate when temperature is expected to be an important driver (e.g. near thresholds) and when it is not (Parham *et al. * 2015).
(8) **Microclimates and habitats vary in temperature.** While studies often focus on constant or mean temperatures, in the field vectors can preferentially occupy habitats with favourable microclimates (Paaijmans *et al. * 2010b; Paaijmans & Thomas 2011b; Murdock *et al. * 2017). How much do microhabitat availability and preferences moderate the effects of average temperatures on transmission?	Collect mosquitoes in their preferred resting and breeding habitats in the field. Experimentally measure traits in different microclimates in the field (Murdock *et al. * 2017). Adapt mechanistic models to incorporate temperatures from relevant microclimates, rather than averages over larger spatial scales.
(9) **Temperature varies over time.** Temperature varies across hours, days, weeks, seasons, and years. Because of Jensen’s inequality, temperature variation has nonlinear effects on trait performance and transmission. What are the most important time scales of temperature variation, and how do they influence transmission?	Adapt approaches for nonlinear averaging (Savage 2004; Bernhardt *et al. * 2018) to understand how temperature variation affects traits and transmission (Paaijmans *et al. * 2010a; Lambrechts *et al. * 2011; Blanford *et al. * 2013). Experimentally test how well nonlinear averaging across thermal performance curves estimated at constant temperatures can predict trait performance at variable temperatures.
(10) **Thermal performance curves vary across individuals, populations, and species.** Trait thermal responses are expected to vary due to genetic variation, phenotypic plasticity (e.g. acclimation), environmental conditions, and chance (Cator *et al. * 2019). How much variation exists, at what scales (individual, population, species), and how does it affect transmission? Are differences in thermal performance curves among species predictable based on phylogenetic, geographic, or ecological factors?	Use physiological models and trait databases to predict variation in thermal responses (Rohr *et al. * 2018; Cator *et al. * 2019). Experimentally measure trait thermal performance curves across different field‐derived populations. In thermal response experiments, measure traits at the individual level to quantify individual trait variation and covariation among traits. Use metabolic theory of ecology to identify systematic differences in trait performance curves that vary with phylogenetic and ecological predictors.
(11) **Time lags between climate and transmission.** Transmission requires multiple developmental processes to occur in the mosquito and parasite, resulting in time lags that vary with temperature. How do these temperature‐dependent lags affect our ability to measure and infer the effects of climate on transmission?	Incorporate time‐ (or temperature‐) dependent biological lags into dynamical models, an ongoing area of mathematical biology research. Quantitatively review statistical studies that estimate time lags between climate and vector or disease dynamics (e.g., Mordecai *et al. * 2017; Lowe *et al. * 2018; Shocket *et al. * 2018), to assess how these lags vary geographically and with climate.
(12) **Relative versus absolute limits on transmission.** Our *R_0_* approach estimates the relative temperature suitability physiological limits for transmission (where *R_0_* = 0) but not the stability of the disease‐free equilibrium (where *R_0_* = 1). Other factors are expected to scale the overall magnitude of transmission (e.g., EIR for malaria in Africa varied between 0 and ~ 700 infectious bites per person per year at optimal temperatures of 25ºC) (Mordecai *et al. * 2013). How much do the precise temperature limits at which *R_0_*> 1 vary with the suitability of other conditions for transmission?	Quantify the absolute temperature dependence of transmission by measuring *R_0_* as a disease invades a novel environment (e.g. for an emerging epidemic like Zika (Duffy *et al. * 2009)) or by estimating a temperature‐dependent force of infection after accounting for susceptible depletion (e.g., Perkins *et al. * 2015). For endemic or seasonally epidemic pathogens, use dynamic transmission models to mechanistically incorporate trait thermal performance curves (e.g., Huber *et al. * 2018).
(13) **Potential for thermal performance curves to adapt to warming temperatures.** Vectors and parasites are expected to respond to selective pressures, including rising temperatures, through plasticity and adaptation. How much can thermal performance curves adapt, and how much have they already adapted, to varying climates?	Experimentally measure trait thermal performance curves across populations originating from different climates (Zouache *et al. * 2014; Ruybal *et al. * 2016). Conduct artificial selection experiments on diverse populations to measure potential for adaptation to changing temperature mean, variance, and extremes. Identify candidate genes for thermal adaptation using conserved genetic regions studied in other species (e.g., *Drosophila* spp.) and study their population genetics in the field.

## Approach

### Insights from trait‐based mechanistic models

Here, we synthesise our previous research that applied a trait‐based modelling approach (Fig. 1) to incorporate the empirical effects of constant temperature on mosquito and parasite traits, and in turn transmission, for a variety of mosquito‐borne pathogen systems (Table 2) (Mordecai *et al.* 2013, 2017; Johnson *et al.* 2015; Shocket *et al.* 2018, 2019; Tesla *et al.* 2018). By comparing previous model results – developed with consistent methodology – we investigate how temperature differentially affects transmission across vector‐borne diseases and examine the implications for climate change. Temperature affects all traits of mosquitoes and pathogens that are tied to biological rates, times, and probabilities via metabolism, including many traits that drive transmission. Transmission is a dynamic, nonlinear process that depends on the density of infected vectors and the availability of susceptible hosts. Time lags arise between climate and transmission because temperature affects mosquito development rates in immature stages, the oviposition cycle, and pathogen extrinsic incubation period (Stewart Ibarra *et al.* 2013; Huber *et al.* 2016). Temperature varies at multiple time scales – daily, seasonally, yearly – that affect these traits in different ways (Paaijmans *et al.* 2010a, 2013). As a result, effects of temperature on transmission are nonlinear, dynamic, and complex at multiple biological scales.

**Table 2:** Thermal optima and limits for temperature‐dependent *R_0_* models across systems

*System*	*Topt* (CIs)	*Tmin* (CIs)	*Tmax* (CIs)
EEEV | *Ae. triseriatus*	22.7 (22.0–23.6)	11.7 (8.8–16.3)	31.9 (31.1–33.0)
WEEV | *Cx. tarsalis*	23.0 (22.0–24.7)	8.6 (6.3–13.0)	31.9 (30.3–35.2)
SINV | *Cx. pipiens*	23.2 (21.7–24.6)	9.4 (6.9–13.3)	33.8 (28.2–37.0)
WNV | *Cx. univittatus*	23.8 (22.7–25.0)	11.0 (8.0–15.3)	33.6 (31.2–36.9)
WNV | *Cx. tarsalis*	23.9 (22.9–25.9)	12.1 (9.6–15.2)	32.0 (30.6–38.6)
SLEV | *Cx. tarsalis*	24.1 (23.1–26.0)	12.9 (11.0–14.8)	32.0 (30.6–38.5)
WNV | *Cx. pipiens*	24.5 (23.6–25.5)	16.8 (14.9–17.8)	34.9 (32.9–37.6)
WNV | *Cx. quinquefasciatus*	25.2 (23.9–27.1)	19.0 (14.1–20.9)	31.8 (31.1–32.2)
*P. falciparum* | *Anopheles*	25.4 (23.9–27.0)	19.1 (16.0–23.2)	32.6 (29.4–34.3)
RVFV | *Ae. taeniorhynchus*	25.9 (23.8–27.1)	10.6 (8.6–14.4)	37.8 (34.4–39.1)
SINV | *Ae. taeniorhynchus*	26.0 (23.9–27.3)	9.7 (8.3–13.6)	37.8 (34.4–39.2)
DENV | *Ae. albopictus*	26.4 (25.4–27.6)	16.2 (13.0–19.8)	31.4 (29.5–34.0)
RRV | *Cx. annulirostris*	26.4 (26.0–26.6)	17.0 (15.8–18.0)	31.4 (30.4–33.0)
MVEV | *Cx. annulirostris*	26.4 (26.2–26.8)	17.0 (16.0–19.2)	31.4 (30.4–33.0)
ZIKV | *Ae. aegypti*	28.9 (28.2–29.6)	22.8 (20.5–23.8)	34.5 (34.1–36.2)
DENV | *Ae. aegypti*	29.1 (28.4–29.8)	17.8 (14.6–21.2)	34.5 (34.1–35.8)

Posterior estimates of mean optimal temperatures, lower thermal limits and upper thermal limits (with 95% credible intervals in parentheses) for each of our temperature‐dependent *R_0_* models. Models are named for the predominant vector and pathogen species from which trait thermal responses were derived, but models occasionally filled in missing traits with the most conservative estimates available from closely‐related species (Mordecai *et al.* 2013, 2017; Johnson *et al.* 2015; Shocket *et al.* 2018; in prep; Tesla *et al.* 2018). The parasite abbreviations are as follows: Eastern equine encephalitis virus (EEEV), Western equine encephalitis virus (WEEV), Sindbis virus (SINV), West Nile virus (WNV), St. Louis encephalitis virus (SLEV), Rift Valley fever virus (RVFV), dengue virus (DENV), Ross River virus (RRV), Murray Valley encephalitis virus (MVEV), and Zika virus (ZIKV). The vectors are abbreviated as *Culex* (*Cx.*) and *Aedes *(*Ae.*).

To capture nonlinearity and complexity in a simple, easily‐used model, we have ignored temperature variation and dynamic time lags (Table 1) in this work and represented the effects of temperature on transmission by focusing on a common summary of transmission, the basic reproduction number *R_0_*, at constant temperatures. This number describes the average number of secondary cases that result from a single infected individual introduced into a fully susceptible population. Critically, *R_0_* can incorporate all the temperature‐dependent mosquito and pathogen traits that control transmission, providing a simple metric for examining emergent, nonlinear effects of temperature. Reproduction numbers can be calculated using multiple methods, with differing interpretations (Heesterbeek 2002; Heffernan *et al.* 2005; Diekmann *et al.* 2009), although their direct application is limited in realistically variable environments (Bacaër & Guernaoui 2006; Bacaër & Ait Dads 2012). Because several non‐temperature factors that vary across transmission settings can influence the absolute magnitude of *R_0_*, our work has focused on the relative temperature suitability for transmission, a version of *R_0_* rescaled from zero to one derived exclusively from temperature relationships. The aim is to identify (constant) temperature limits on transmission (where *R_0_* = 0, so transmission is impossible) and the optimal temperature for transmission (where *R_0_* peaks) as metrics that can be compared across mosquito–pathogen systems, transmission settings and climate scenarios. Temperature variation can then be incorporated into these models in the future (see Table 1 and *Limitations to the R_0_(T) approach*).

This approach begins with a commonly used derivation based on dynamical susceptible–infected–recovered (SIR) models and leading‐eigenvalue calculation (Dietz 1993), an extension of the Ross–Macdonald framework (Smith *et al.* 2007b; Reiner *et al.* 2013):

(1) $$R_{0}\left(T\right)=\sqrt{\frac{a{\left(T\right)}^{2}b\left(T\right)c\left(T\right)\exp\left(-\mu \left(T\right)/PDR\left(T\right)\right)M\left(T\right)}{Nr\mu \left(T\right)}},$$

where *r* is the host recovery rate, *N* is the density of hosts, and *T* indicates the temperature dependence of the following parameters (traits): mosquito biting rate, *a*; mosquito adult mortality rate, *μ* (the inverse of lifespan, *lf*); parasite development rate, *PDR* (the inverse of the extrinsic incubation period); and vector competence (the product of the proportion of exposed mosquitoes that acquire a disseminated infection, *c* and the proportion of infected mosquitoes that become infectious with pathogens in their salivary glands, *b*).

Temperature should also affect mosquito abundance, *M*, because it affects mosquito life‐history traits. Parham & Michael (2010) extended eqn (1) to incorporate effects of temperature on mosquito abundance via its effects on egg‐to‐adult development rate (*MDR*) and survival probability (*p_EA_*), and lifetime fecundity (*B*, which we approximate as eggs per female per day, *EFD*, times lifespan, 1/*μ*). Following Parham & Michael (2010), our work has modelled temperature‐dependent mosquito abundance as follows:

(2) $$M\left(T\right)=\frac{EFD\left(T\right)p_{\mathrm{EA}}\left(T\right)MDR\left(T\right)}{\mu {\left(T\right)}^{2}}$$

Incorporating *M*(*T*) into the *R_0_*(*T*) model, we obtained the full temperature‐sensitive *R_0_* expression (Mordecai *et al.* 2013, 2017; Johnson *et al.* 2015; Shocket *et al.* 2018; Tesla *et al.* 2018):

(3) $$R_{0}\left(T\right)=\sqrt{\frac{a{\left(T\right)}^{2}b\left(T\right)c\left(T\right)\exp\left(-\mu \left(T\right)/PDR\left(T\right)\right)EFD\left(T\right)p_{\mathrm{EA}}\left(T\right)MDR\left(T\right)}{Nr\mu {\left(T\right)}^{3}}}$$

To estimate the effect of temperature on *R_0_*, in previous work, we parameterised thermal response functions for each of the temperature‐dependent parameters using laboratory experiments that measure mosquito or pathogen traits at three or more constant temperatures. Based on data availability, for some mosquito species, we used alternative measurements of immature survival and fecundity (Table S1) and adjust eqn (3) accordingly (Shocket *et al.* 2019).

This approach is simple, mechanistic, analytical and broadly applicable across the vector‐borne disease systems for which eqns ((1), (2), (3)) apply (i.e. mosquito‐ and fly‐borne pathogens). In this framework, we have used sensitivity analyses to examine the effects of different temperature‐dependent traits on transmission, and Bayesian inference to assess how uncertainty in traits affects uncertainty in *R_0_* across temperatures, pinpointing critical traits and temperatures for further data collection (Johnson *et al.* 2015). We have applied this approach to a variety of mosquito vectors and pathogens to estimate temperature‐dependent transmission functions that can be field‐tested and compared across systems (Mordecai *et al.* 2013, 2017; Shocket *et al.* 2018, 2019; Tesla *et al.* 2018) (Table 2).

### *Limitations to the R_0_*(*T*) *approach*

This approach has several important limitations, some of which can be addressed by extending the models – making them less general – while others represent priorities for future research (summarised in Table 1). One limitation of constant‐temperature models is that in nature temperature varies daily, seasonally, interannually, and spatially. Because trait thermal responses are nonlinear, trait performance under varying temperatures deviates from performance at constant temperatures due to Jensen’s inequality (Table 1) (Martin & Huey 2008; Paaijmans *et al.* 2010a; Lambrechts *et al.* 2011). Performance at variable temperatures exceeds performance at a constant temperature for concave‐up regions of thermal performance curves (near thermal limits), and vice versa for concave‐down regions of thermal performance curves (near thermal optima) (Bernhardt *et al.* 2018). For example, parasites may complete development within the mosquito under varying temperatures centred on constant temperatures at which development would never be completed, near thermal limits (Blanford *et al.* 2013). Because of nonlinear thermal performance curves, the optimal mean temperature in a variable environment depends on the amount of temperature variation (Martin & Huey 2008; Paaijmans *et al.* 2010a; Lambrechts *et al.* 2011; Blanford *et al.* 2013). Future work could incorporate variance and higher‐order terms for fluctuation in temperature across different time scales to estimate its effect on transmission (Table 1) (Savage 2004; Cohen *et al.* 2019).

Nonlinearities also make many traits difficult to measure even at constant temperatures, especially near thermal limits (Table 1). For cold‐skewed thermal performance curves, trait performance can drop steeply from peak to zero over a few degrees, so experiments must span wide ranges of temperatures in small increments to fully capture nonlinear thermal responses. Rates can be difficult to measure near lower thermal limits because they are exceedingly slow (Waite *et al.* 2019). Near upper performance limits, constant temperature estimates may not capture non‐lethal and time‐dependent effects of heat stress (Kingsolver & Woods 2016; Sinclair *et al.* 2016). Traits are most easily and accurately measured near thermal optima, while uncertainty is highest near thermal limits (Johnson *et al.* 2015). For composite traits such as vector competence, measuring the underlying physiological responses may be important for understanding thermal responses, particularly under varying temperatures (Table 1).

Keeping these empirical limitations in mind, we have applied *R_0_*(*T*) estimated from constant‐temperature trait thermal performance experiments as a consistent metric of relative temperature suitability across vector‐borne diseases. This metric captures the emergent, nonlinear effects of temperature on disease transmission, allowing us to investigate general patterns in thermal responses. The models could be extended to include temperature variation, individual‐ or population‐level trait variation, differences in performance between the laboratory and the field, and mosquito and human behaviour (Table 1), gaining explanatory power while losing generality.

## Synthesising previous results

**Figure 2:**
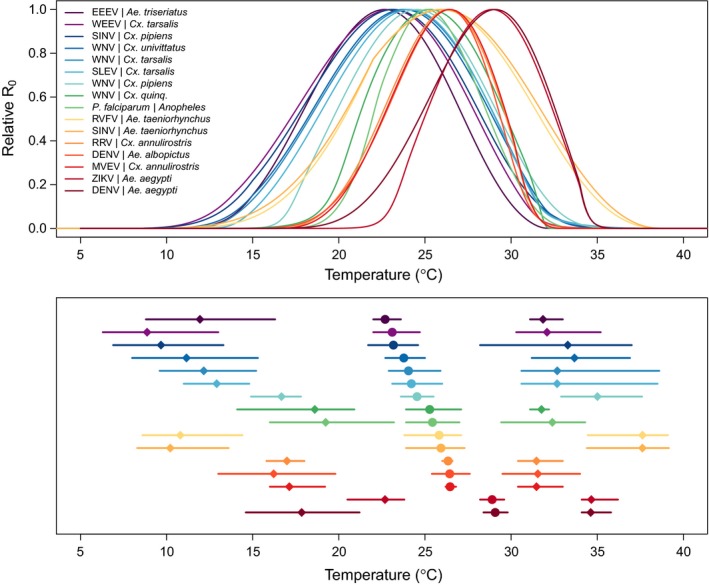
Temperature‐dependent *R_0_* models are consistently unimodal with differing thermal optima and limits across systems. Top panel: temperature‐dependent *R_0_* models for 16 vector–pathogen systems; bottom panel: *R_0_* thermal optima (temperature where *R_0_* peaks; circles) and lower and upper limits (temperature where *R_0_* = 0; diamonds), with 95% credible intervals (lines). Curves depict empirically parameterised temperature‐dependent *R_0_* models for each vector – pathogen pair, normalised so the y‐axis ranges from 0 to 1 because other factors that affect the absolute magnitude of *R_0_* vary by system. Colors designate different vector – pathogen systems, ordered by thermal optima for *R_0_* in the both panels. Abbreviations for all vectors and parasites are given in Table S1.

**Figure 3:**
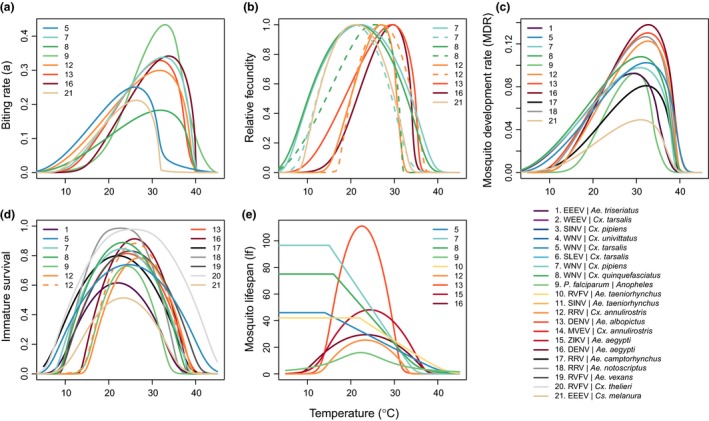
Trait thermal performance curves for vector life history traits vary by species. Thermal performance curves estimated from laboratory experimental data across different vector species that transmit different pathogens (systems are numbered in each panel; overall system numbering key and color scheme are in the main legend). Color scheme is consistent with Fig. 2, i.e., ordered by thermal optima for *R_0_*; systems for which no *R_0_* model was calculated are listed last. Vector traits are (a) biting rate (*a*); (b) relative fecundity; (c) mosquito development rate (*MDR*); (d) immature survival; and (e) mosquito lifespan (*lf*). Fecundity is rescaled to range from zero to one because it is alternatively measured as eggs per female per day (*EFD*; *Ae. aegypti, Cx. annulirostris*), eggs per female per oviposition cycle (*EFOC*; *Ae. albopictus, Cx. pipiens*), number of larvae per raft (*n_LR_*; *Cx. annulirostris* [dashed line]), eggs per raft (*EPR*; *Cx. quinquefasciatus*), or proportion ovipositing (*p_O_*; *Cx. pipiens* [dashed line], *Cx. quinquefasciatus* [dashed line], *Cs. melanura*). Immature survival probability is measured as egg‐to‐adult survival probability (*p_EA_*; *Ae. aegypti, Ae. albopictus, An. gambiae*), larva‐to‐adult survival probability (*p_LA_*; *Ae. camptorhynchus, Ae. notoscriptus, Ae. triseriatus, Ae. vexans, Cx. annulirostris, Cx. pipiens, Cx. quinquefasciatus, Cx. tarsalis, Cs. melanura*), proportion of egg rafts that hatch (*p_RH_*; *Cx. annulirostris* [dashed line]), or egg viability (*EV*; *Cx. thelieri*). To be conservative, for three temperate vectors that can undergo diapause and therefore survive cold temperatures (*Cx. tarsalis, Cx. pipiens, Cx. quinquefasciatus*), lifespan (*lf*) was assumed to be constant from 0ºC to the lowest temperature measured in the experiments (14‐16ºC), because a decline at low temperatures was not observed in the data. Abbreviations for all vectors and parasites are given in Table S1.

**Figure 4:**
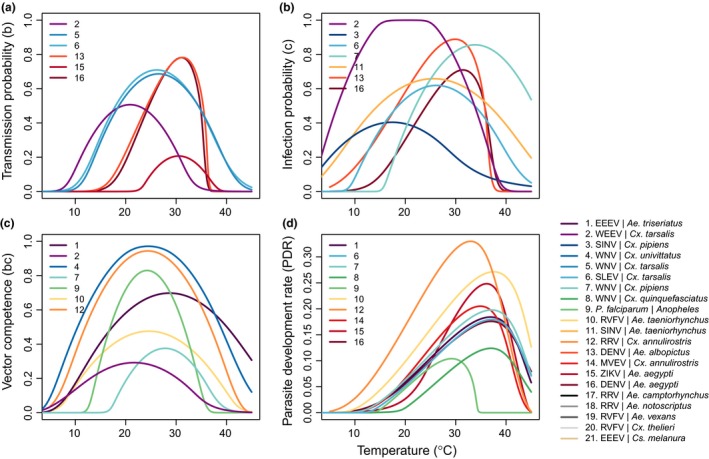
Trait thermal performance curves for pathogen transmission traits within the vector vary by species. Thermal performance curves estimated from laboratory experimental data across different pathogens and vectors (systems are numbered in each panel; overall system numbering key and color scheme are in the main legend). Color scheme is consistent with Figs 2 and 3. Traits are (a) transmission probability (*b*); (b) infection probability (*c*); (c) vector competence (*bc*); and (d) parasite development rate (*PDR*). Abbreviations for all vectors and parasites are given in Table S1.

**Figure 5:**
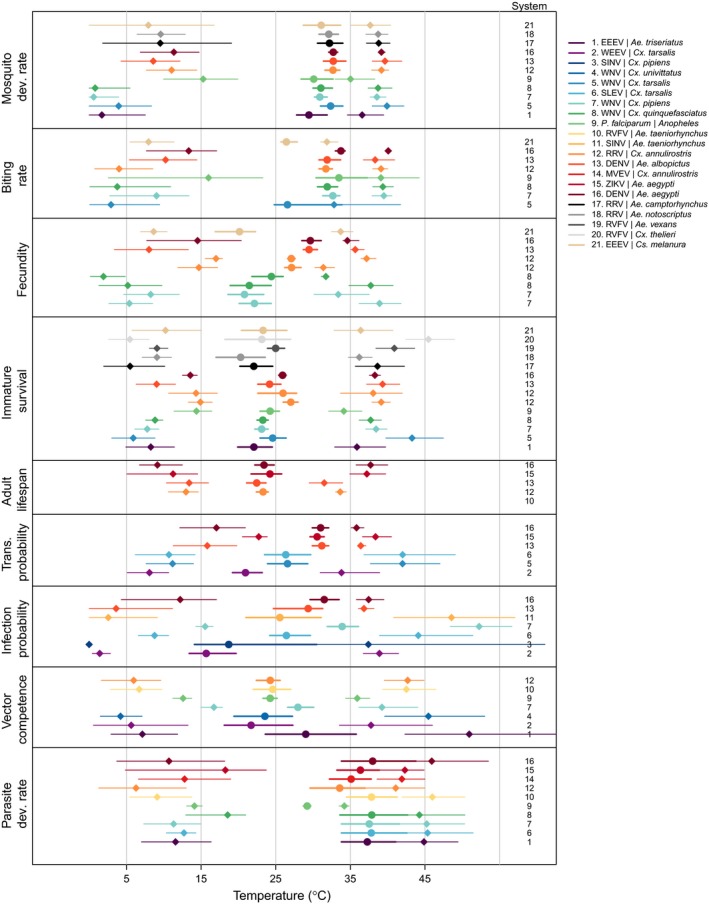
Traits vary in thermal minimum, optimum, and maximum across species. For each vector and/or pathogen for which a trait was measured, points show the mean thermal optimum (circles) and lower and upper thermal limits (diamonds) along with their 95% credible intervals (lines). Traits are mosquito development rate (*MDR*), biting rate (*a*), fecundity (*EFD, EFOC, n_LR_, EPR, p_O_*), immature survival (*p_EA_, p_LA_, p_RH_, EV*), adult mosquito lifespan (*lf*), transmission probability (*b*), infection probability (*c*), vector competence (*bc*), and parasite development rate (*PDR*). Traits for which minima, optima, or maxima were not estimated are not shown. Not all traits were measured in all species. Color scheme is consistent with Figs 2 and 3. Systems are numbered to the right of each trait; overall system numbering key and color scheme are in the main legend. Abbreviations for all vectors and parasites are given in Table S1.

We empirically parameterised thermal performance curves for traits and *R_0_* for a suite of 15 ecologically important mosquito species that transmit 11 different pathogens: Western and Eastern Equine Encephalitis virus (WEEV and EEEV), Sindbis virus (SINV), Rift Valley Fever virus (RVFV), West Nile virus (WNV), St. Louis Encephalitis virus (SLEV), *Plasmodium falciparum* malaria, Ross River virus (RRV), Murray Valley Encephalitis virus (MVEV), dengue virus (DENV) and Zika virus (ZIKV) (Figs 2, 3, 4, 5; Table 2, Table S1). In total, we empirically estimated thermal performance curves for 88 traits, resulting in 16 temperature‐dependent *R_0_* models (in some cases, we estimated separate *R_0_* models for different pathogens in the same vector or different vectors for the same pathogen) (Mordecai *et al.* 2013, 2017; Johnson *et al.* 2015; Shocket *et al.* 2018, 2019; Tesla *et al.* 2018). With these thermal performance curves, we compare temperature responses across vector–pathogen systems and estimate potential effects of climate change on disease transmission.

As expected, all of the mosquito and pathogen traits we examined were temperature‐sensitive and generally respond unimodally, peaking from 15.7 to 38.0°C (mean thermal optimum = 28.4°C; Figs 3, 4, 5). (One exception is lifespan for three temperate mosquitoes, discussed below; Fig. 3.) These traits were estimated to decline to zero at thermal minima ranging from 0 to 22.7°C (mean = 9.5°C) and maxima ranging from 31.4 to 56.6°C (mean = 39.5°C; Fig. 5). Mosquito immature survival and adult lifespan had the lowest thermal optima, while pathogen development rate had the highest thermal optima (Fig. 5). Vector competence often peaked at much lower temperatures than pathogen development rate (Fig. 5), emphasising the importance of measuring both trait thermal responses (Paaijmans *et al.* 2011). Temperate mosquito species had notably higher immature survival at temperatures below 10°C than the sub‐tropical and tropical species (Fig. 3), suggesting that this trait is important for persistence in temperate climates. Consistent with previous ectotherm physiology work (Amarasekare & Savage 2012), rate traits usually exhibited asymmetric unimodal thermal responses with higher optima, while probability traits were more symmetrical with lower optima (Figs 3 and 4). Asymmetric curves for rates such as immature development, fecundity, biting, and parasite incubation can be well fit by Brière and modified Arrhenius functions, while symmetrical curves for probability traits such as survival and vector competence are well described by quadratic or Gaussian functions (Figs 3 and 4) (Johnson & Lewin 1946; Briere *et al.* 1999; Amarasekare & Savage 2012; Molnár *et al.* 2013, 2017).

In contrast to the unimodal responses for other traits, the lifespans of three temperate mosquitoes – *Culex pipiens*, *Cx. tarsalis*, and *Cx. quinquefasciatus* – monotonically declined across the temperature ranges measured (above 14°C). These species may differ in their biological responses to low temperature because of their ability to diapause as an adaptation to survive cold winters (Shocket *et al.* 2019). Further research in species like *Ae. albopictus*, which differ geographically in the ability to diapause, could test the hypothesis that diapause affects the shape of the thermal performance curve for lifespan (Table 1) (Thomas *et al.* 2012). Nonetheless, theory predicts that exposure to more extreme cold temperatures would eventually limit lifespans even for diapausing mosquitoes, suggesting a unimodal response over a wider temperature range.

Synthesising the overall influence of temperature on disease transmission (*R_0_*), we found several commonalities in the thermal performance curves and thermal limits from our trait‐based models. First, we estimated that transmission peaked at intermediate temperatures between 22.7 and 29.1°C (mean optimal temperature = 25.2°C; Fig. 2). Lower thermal limits for modeled *R_0_* under constant temperatures ranged from 8.7 to 22.7°C and upper thermal limits ranged from 31.5 to 37.8°C (Fig. 2). As expected, several temperate mosquito species had relatively cool thermal ranges for transmission, although the lower thermal limits are more divergent than the thermal optima and upper limits (Fig. 2; Table 2). Tropical mosquito‐borne diseases had intermediate thermal optima within the environmentally relevant range of temperatures, ranging from 25.4°C for *P. falciparum* malaria in *Anopheles gambiae* to 29.1°C for DENV in *Ae. aegypti*. At the same time, many temperate vectors and pathogens (e.g., *Cx. pipiens*, *Cx. tarsalis*, and *Cx. quinquefasciatus* and WNV, EEEV, WEEV, SLEV, and SINV), had *R_0_* thermal limits up to 32–35°C, similar to those of tropical diseases (Fig. 2; Shocket *et al.* 2019). Finally, the temperature response of *R_0_* – particularly the lower thermal limit and optimum – varied across multiple pathogens transmitted by a single mosquito species (e.g. DENV and ZIKV in *Ae. aegypti*; WNV, WEEV, and SLEV in *Cx. tarsalis*; WNV and SINV in *Cx. pipiens*) and across multiple mosquito species transmitting a single pathogen (e.g. DENV in *Ae. albopictus* and *Ae. aegypti*; WNV in *Cx. tarsalis*, *Cx. pipiens*, *Cx. quinquefasciatus*, and *Cx. univittatus*) (Fig. 2; Table 2). This implies that the thermal response of transmission depends on the effects of temperature on traits of both the mosquito and the pathogen.

Sensitivity analyses conducted in previous work showed that all mosquito and pathogen traits limit the thermal response of transmission – including traits regularly incorporated into temperature‐dependent models like mosquito lifespan, extrinsic incubation rate, and biting rate as well as less‐recognised temperature‐dependent traits like vector competence and demographic rates (Mordecai *et al.* 2013, 2017; Johnson *et al.* 2015; Shocket *et al.* 2018; Tesla *et al.* 2018). Adult mosquito lifespan consistently limited *R_0_* at warm temperatures while pathogen development rate (i.e., extrinsic incubation rate) and mosquito biting rate limited *R_0_* at low temperatures (Mordecai *et al.* 2013, 2017; Shocket *et al.* 2018, 2019). However, in some systems other traits determined lower and upper thermal limits, and optima for transmission varied across systems. For RRV in *Cx. annulirostris*, fecundity and immature survival determined the upper and lower limits, respectively (Shocket *et al.* 2018). For WNV in *Cx. pipiens*, vector competence determined the lower limit (Shocket *et al.* 2019). For WNV in *Cx. quinquefasciatus*, oviposition rate determined the upper limit, and for WNV, WEEV, and SLEV in *Cx. tarsalis*, biting rate was important for the upper limits (Shocket *et al.* 2019). Because *R_0_* models were sensitive to different traits for different systems, it is critical to include empirically‐estimated thermal responses for the full suite of traits in transmission models. Models based on more limited thermal response assumptions often predict very different effects of temperature on transmission, which align poorly with field data.

## Discussion

### Strengths and limitations of mechanistic and statistical approaches

Research on the effects of temperature on mosquito‐borne disease has taken either mechanistic (trait‐based) or statistical (correlative) approaches (Rogers & Randolph 2006). Mechanistic approaches are most suitable for characterising the multivariate, nonlinear effects of temperature on disease transmission across broad temperature and geographic ranges, for estimating the fundamental environmental limits on transmission, and for extrapolating into future climate regimes and novel settings, for which statistical approaches have limited applicability (Parham *et al.* 2015). However, statistical models are often more appropriate for locally predicting disease dynamics, for describing or forecasting transmission over smaller spatial or temporal scales, for understanding the relative explanatory power of multiple drivers of transmission, and for describing current patterns of disease transmission (Parham *et al.* 2015).

Mechanistic models rely on our understanding of the dominant processes that underlie organismal fitness to predict geographic range, population size, or transmission rate (Helmuth *et al.* 2005; Kearney *et al.* 2009, 2010; Kearney & Porter 2009), and therefore require experiments on multiple traits of pathogens, vectors, and their interactions across environmental conditions. Synthesising heterogeneous data sources from the literature, including filling in parameter values from related species where necessary (e.g., Mordecai *et al.* 2013), introduces methodological and biological differences that can add error to parameter estimates. Unlike statistical approaches, such as species distribution models (SDMs), mechanistic models often do not jointly capture many biotic and abiotic constraints that limit observed ranges.

The value of mechanistic models is their flexibility and extensibility, which are particularly important for predicting distributions and dynamics in novel environments (Williams & Jackson 2007). Because experiments can capture mechanistic responses to both current and anticipated climatic conditions, they are more appropriate than SDMs for predicting species responses to novel or non‐equilibrium contexts, such as climate change (Helmuth *et al.* 2005; Kearney & Porter 2009). Moreover, many statistical models have several key limitations: limited geographic and/or temporal extent (Table S2), limited capacity to separate effects of multiple interacting and covarying environmental, population, and behavioural factors, and the assumption that the processes currently limiting ranges have set range limits in the past and will continue to do so in the future, despite entering unprecedented regimes of climate, population movement, and interventions. Direct comparisons of statistical and mechanistic models revealed that they performed similarly at predicting current distributions, but predicted differential responses of species to a uniform warming (Buckley *et al.* 2010).

These approaches are complementary because of their differing strengths, applications, and data requirements (Parham *et al.* 2015). Thermal biology principles that govern mosquito‐borne disease transmission – i.e., that temperature, and therefore climate change, has nonlinear effects on multiple traits and on transmission as a whole – apply to both mechanistic and statistical approaches.

### Comparing our results with other mechanistic models

The assumption that warmer, wetter conditions universally promote mosquito‐borne disease is pervasive in the literature (Ermert *et al.* 2012; Morin *et al.* 2015; Caminade *et al.* 2017; Thomson *et al.* 2017). Yet our trait‐based work (Figs 2, 3, 4, 5) has highlighted the importance of applying rigorous thermal biology in mosquito‐borne disease models to accurately estimate nonlinear effects of temperature. Many models assume that only mosquito longevity, extrinsic incubation period, and sometimes biting rate are temperature‐sensitive, measure either temperature‐dependent vector competence or extrinsic incubation period but not both, and/or assume monotonic thermal responses that do not account for unimodal effects of temperature (e.g. Martens *et al.* 1997; Craig *et al.* 1999; Fros *et al.* 2015; Morin *et al.* 2015; Perkins *et al.* 2016; Vogels *et al.* 2016; Paull *et al.* 2017; Siraj *et al.* 2017). Even when they predict that the effects of temperature on transmission are unimodal overall, these models with limited thermal biology often predict very different thermal optima and limits for transmission than models that include the full suite of nonlinear trait thermal responses (Table 3).

**Table 3:** Thermal optima and limits vary substantially across previous mechanistic models of vector‐borne disease transmission

System	Topt	*T*_min_	*T*_max_	Study
**Falciparum malaria (*Anopheles* spp.)**	**25**	**16**	**34**	Mordecai *et al. *(2013)
Falciparum malaria (*Anopheles* spp.)	31	18	38	Martens *et al. *(1997)
Falciparum malaria (*Anopheles gambiae*)	30	18	40	Craig *et al. *(1999)
Falciparum malaria (*Anopheles* spp.)	32–33	20	39	Parham & Michael (2010)
Falciparum malaria (*Anopheles stephensi*)	29	12	38	Shapiro *et al. *(2017)
**DENV, CHIKV, ZIKV (*Ae. aegypti*)**	**29**	**18**	**35**	Mordecai *et al. *(2017)
**DENV, CHIKV, ZIKV (*Ae. albopictus*)**	**26**	**16**	**32**	Mordecai *et al. *(2017)
CHIKV (*Ae. aegypti*)	30	–	–	Johansson *et al. *(2014)
DENV (*Ae. aegypti*)	29	12	32	Liu‐Helmersson *et al. *(2014)
DENV (*Ae. aegypti*)	35	21	36	Morin *et al. *(2015)
DENV (*Ae. aegypti*)	29	13	33	Wesolowski *et al. *(2015)
ZIKV (*Ae. aegypti*)	36	–	–	Caminade *et al. *(2017)
ZIKV (*Ae. albopictus*)	29	–	–	Caminade *et al. *(2017)
DENV (*Ae. aegypti*)	33	–	–	Siraj *et al. *(2017)
ZIKV (*Ae. aegypti*)	29	23	35	Tesla *et al. *(2018)
**WNV (*Cx. pipiens*)**	**25**	**17**	**35**	Shocket *et al., *(2019)
**WNV (*Cx. tarsalis*)**	**24**	**12**	**32**	Shocket *et al., *(2019)
**WNV (*Cx*.* quinquefasciatus*)**	**25**	**19**	**32**	Shocket *et al. *(2019)
WNV (*Aedes* and *Culex* spp)	35	18	*	Kushmaro *et al. *(2015)
WNV (*Cx*.* tarsalis, Cx*.* pipiens, Cx*.* quinquefasciatus*)	24‐25	15‐17	–	Paull *et al. *(2017)
WNV (*Cx*.* pipiens*, biotypes *pipiens, molestus*, hybrid)	28	18	*	Vogels *et al. *(2017)

For each vector‐borne disease and study, estimated thermal optima (*Topt*) and lower and upper thermal limits (*T*_min_ and *T*_max_, respectively) in degrees Celsius were taken from the original papers or by plotting the models. Models include *R_0_*, vectorial capacity, and other related measures of transmission. Dashes (‐) indicate that thermal limits were not reported. Asterisks (*) indicate that models were not unimodal. Models presented in this paper (Table 2) are shown in bold for comparison. The parasite abbreviations are as follows: dengue virus (DENV), chikungunya virus (CHIKV), Zika virus (ZIKV), and West Nile virus (WNV). Note: Johnson *et al. *(2015) used Bayesian inference refit the models for falciparum malaria in *Anopheles* spp. from the original formulation in Mordecai *et al. *(2013) and reported similar *Topt*, *T*_min_ and *T*_max_ estimates of approximately 25, 19, and 33 degrees Celsius, respectively, depending on the choice of priors.

For falciparum malaria, our published *R_0_* model predicted a suitable temperature range of 17–34ºC and an optimum of 25ºC (Mordecai *et al.* 2013; Johnson *et al.* 2015), while earlier models with more limited thermal biology assumptions predicted 3–15ºC wider temperature ranges and 5–7ºC higher optimal temperatures (Table 3) (Martens *et al.* 1997; Craig *et al.* 1999; Parham & Michael 2010). Independent field data on the number of malaria‐infectious mosquitoes per person per year (entomological inoculation rate; EIR) across Africa strongly support our predicted optimum of 25ºC and declines in transmission above 28ºC, providing little support for previous predictions (Fig. S1) (Mordecai *et al.* 2013). Similarly, for dengue, chikungunya, and Zika viruses in *Ae. aegypti*, previous models with more limited thermal biology assumptions predicted thermal optima for transmission up to 6ºC higher than our published *R_0_* model, which peaked at 29ºC (Table 3) (Johansson *et al.* 2014; Liu‐Helmersson *et al.* 2014; Morin *et al.* 2015; Wesolowski *et al.* 2015; Caminade *et al.* 2017; Siraj *et al.* 2017). We found a strong positive relationship between predicted temperature‐dependent *R_0_* and human incidence of dengue (> 85% accuracy) and chikungunya and Zika (> 66% accuracy) across the Americas in 2014–2016 (Fig. S2) (Mordecai *et al.* 2017). From a more recently published version of our *Ae. aegypti R_0_* model updated with Zika‐specific traits (which also peaked at 29ºC), 71.5% of cases in Colombia fell within municipalities with 1–12 months of predicted temperature suitability (mismatches were often due to spatial grain of the data), strongly supporting our predicted relationship (Tesla *et al.* 2018). Finally, for West Nile virus, previous models predicted thermal optima up to 11ºC higher than our predicted optima of 24–25ºC (Table 3) (Kushmaro *et al.* 2015; Paull *et al.* 2017; Vogels *et al.* 2017), which matched the unimodal thermal response of human neuroinvasive West Nile incidence that also peaked at 24ºC (Fig. S3; Shocket *et al.* 2019).

Together, these examples illustrate the importance of both accurately incorporating a full suite of empirically derived, nonlinear trait thermal responses into mechanistic models and validating the models against field data. Other published mechanistic models did not directly report the inferred relationship between temperature and transmission nor validate the relationship with independent field data (e.g., Brady *et al.* 2014; Perkins *et al.* 2016; Li *et al.* 2019), making model comparison difficult.

### Model validation

Connecting mechanistic model predictions to independent field data – model validation – is critical for comparing different models and for assessing their applicability in the field (Hooten & Hobbs 2015). Although several potential approaches exist, including simulating data from mechanistic models to compare with observed vector abundance or case incidence (e.g., Morin *et al.* 2015; Kramer *et al.* 2016), or testing the accuracy of models fit to a training dataset when predicting a separate testing dataset (e.g. Smith *et al.* 2007a; Ren *et al.* 2016), few studies have applied existing methods to validate vector‐borne disease models (but see Tompkins & Ermert 2013; Wesolowski *et al.* 2015).

Several challenges have limited model validation (Table 1). First, the relationship between *R_0_* and incidence of human cases – the most commonly available data for validation – is nonlinear (Smith *et al.* 2007b), so their similarity may be difficult to assess. Second, in local time series data, temperature ranges may cover only a subset of globally relevant temperatures and covary with other environmental factors, but larger‐scale datasets that span a wider range of temperatures introduce error from spatial aggregation and confounding variation in other factors that affect transmission. Third, because environmental drivers affect transmission at varying time lags, the time scales on which to compare temperature and transmission are not obvious. Validation of mechanistic temperature‐dependent transmission models therefore remains a critical gap in theory, statistical approaches, and data availability (Table 1).

In light of these challenges, we have taken several approaches to validating mechanistic models, depending on the type and scale of data available. First, we graphically compared predicted temperature‐dependent *R_0_* with entomological inoculation rate (EIR, a metric closely related to *R_0_* (Smith *et al.* 2007b)) for malaria plotted against average transmission season temperature from data spanning 30 years across Africa (Hay *et al.* 2000). We showed that the maximum EIR within data subsets binned by temperature were closely correlated with predicted *R_0_*, though EIR varied greatly within temperature bins (Fig. S1) (Mordecai *et al.* 2013). With incidence data, we graphically compared seasonal and geographical patterns with predicted temperature‐dependent *R_0_*. Ross River virus incidence in Australia from 1992 to 2013 closely aligned with predicted average seasonal temperature‐dependent *R_0_* across cities weighted by population size, with a 2‐month lag (Fig. S4) (Shocket *et al.* 2018). As described above, our mechanistic model predictions corresponded closely with monthly county‐level West Nile neuroinvasive disease incidence in the US from 2001 to 2016 (Fig. S3) (Shocket *et al.* 2019), weekly dengue, chikungunya, and Zika incidence across countries in the Americas from 2014 to 2016 (Fig. S2) (Mordecai *et al.* 2017), and Zika occurrence across municipalities in Colombia from 2015 to 2017 (Tesla *et al.* 2018). Together, this diverse set of field data and approaches shows that temperature‐dependent *R_0_* is often strongly associated with observed patterns of disease, despite the known limitations of the models and data.

### Comparison with previous statistical models

In contrast to mechanistic models, statistical models have directly inferred relationships between climate and vector abundance, occurrence, or disease incidence from field data at local, regional, and global scales. In particular, species distribution models (SDMs) use statistical analyses of geographic records of vector or disease occurrence and climate and other environmental covariates to predict species geographic distributions and their ecological determinants (Table S2). These methods are appealing because they can infer current climate relationships using presence‐only data from health departments or surveillance records, along with remotely sensed or ground‐based climate data.

Most SDMs for vectors and vector‐borne diseases find at least one aspect of temperature (e.g., mean, range, variability) to be an important predictor of occurrence (Table S2). Differences among models may arise because of the difficulty of inferring nonlinear, dynamic effects of temperature in noisy data and the limited range of environmental conditions represented in many studies. Direct model comparison with mechanistic models is not feasible because most SDMs do not directly report either the inferred relationships between climate variables and probability of occurrence or the occurrence probabilities as spatially explicit datasets. This is an important problem because individual SDMs are often difficult to reproduce, externally validate, or apply to new research settings. Without such improvements (Qiao *et al.* 2016; Sloyer *et al.* 2019), the majority of published SDMs cannot contribute substantially to our understanding of the drivers and projected changes in vector‐borne disease transmission. Recent efforts in model comparison and ensemble modelling for mosquito‐borne disease (Yamana *et al.* 2016; Little *et al.* 2017; Carlson *et al.* 2018) highlight how multi‐model synthesis can move the field forward when model assumptions and results are transparent and reproducible. Nonetheless, SDMs provide evidence that temperature is a strong statistical predictor of the occurrence of vector‐borne diseases and vectors, supporting principles from vector thermal biology.

### Evidence of unimodal temperature responses in the field

A key principle of thermal biology, supported by mechanistic models (Fig. 2), is that the effects of temperature on mosquito‐borne disease transmission are unimodal. This implies that relationships observed across more limited temperature ranges are expected to range from negative to positive to null. Field‐based empirical support for positive temperature – disease relationships is widespread (e.g., Alonso *et al.* 2011; Mena *et al.* 2011; Stewart‐Ibarra & Lowe 2013; Siraj *et al.* 2014; Lowe *et al.* 2018), but support for unimodal responses or declines at high temperatures is more limited. However, emerging field evidence supports unimodal relationships with temperature, including declines at high temperatures, for dengue incidence and *Aedes aegypti* abundance in Colombia (Peña‐García *et al.* 2016, 2017), for chikungunya incidence in the Americas (Perkins *et al.* 2015), for malaria incidence in Kenya and across Africa (Mordecai *et al.* in review; Shah *et al. * in press), for West Nile disease in the United States (Fig. S3; Shocket *et al.* 2019), and for the vectors of malaria, dengue, chikungunya, yellow fever, Zika, leishmaniasis, and Chagas disease in Ecuador (Escobar *et al.* 2016). Ambiguous or spatially variable relationships between temperature and incidence (e.g., for Ross River virus, Gatton *et al.* 2005; Bi *et al.* 2009; Hu *et al.* 2010; Werner *et al.* 2012; Koolhof *et al.* 2017) may be explained by unimodal thermal responses. In other cases, for example, for malaria in sub‐Saharan Africa, effects of climate are attributed to temperature in cooler, highland areas and to drought in warm, semi‐arid regions, without recognising that high temperatures alone could limit transmission (Ermert *et al.* 2012; Thomson *et al.* 2017). However, recent work has shown negative effects of locally high temperatures on urban malaria transmission in a semi‐arid city in India (Santos‐Vega *et al.* 2019). Mechanistic temperature‐based models are important for predicting effects on disease when climate change alters the covariation among temperature, humidity, rainfall, and drought. We find growing support for unimodal relationships between mosquito‐borne disease and temperature in the field from the few studies that have investigated nonlinear responses, supporting thermal biology principles and mechanistic model predictions (Fig. 2).

### Implications and predictions for climate change

Our synthesis of previous research on the effects of temperature on mosquito‐borne disease transmission provides a clear set of case studies to support the universal importance of nonlinear thermal responses for vector‐borne diseases more generally. From these case studies, we derive four key implications for climate change:

1. Changes in temperature may locally increase, decrease, or have no effect on transmission (Fig. 2). The direction and relative magnitude of these effects are predictable from mechanistic models (Fig. 1) but would otherwise appear idiosyncratic.
2. Accurately estimating the thermal optima and limits for transmission is critical for predicting effects of temperature change on transmission. Models derived from incomplete assumptions for mosquito and pathogen thermal biology can incorrectly estimate both the direction and magnitude of effects of climate change (Table 3).
3. Enhanced climate data products and projections (Thomson *et al.* 2006, 2011, 2017; Caminade *et al.* 2014; Tjaden *et al.* 2018) are only as valuable as the models that link climate to disease transmission, which vary widely in their assumptions, predicted relationships, and field validation (Tables 2‐3).
4. Climate warming may increase the geographic and seasonal ranges of mosquito‐borne diseases with high thermal optima and upper limits relative to their current distributions, including Ross River, dengue, and Zika viruses (Mordecai *et al.,* 2017; Shocket *et al.* 2018; Tesla *et al.* 2018; Ryan *et al.* 2019). However, climate warming is more likely to shift or contract the geographic and seasonal ranges of diseases with lower thermal optima and upper limits, including malaria and West Nile virus (Mordecai *et al.* 2013; Johnson *et al.* 2015; Ryan *et al.* 2015, 2019; Shocket *et al.* 2019).

The trait‐based thermal biology approach illustrated here for mosquito‐borne diseases can be applied more broadly to understand the effects of climate change on many other types of vector‐borne diseases, including plant diseases transmitted by aphids, flies, and psyllids (Taylor *et al.* 2016, 2018) and human and livestock diseases transmitted by biting midges, fleas, and flies (Akey *et al.* 1978; Carpenter *et al.* 2011; Moore *et al.* 2012; Alsan 2015), and tick‐borne diseases (Ostfeld & Brunner 2015; Cheng *et al.* 2017). However, differences in vector and host biology, including the effects of vector life history and biting behaviour and how they interact with host activities and life cycles, can cause major differences between the models presented here (eqns (1), (2), (3)) and the transmission models most appropriate for other systems. For example, in some tick‐borne disease systems the most important effects of climate on human disease risk arise from effects on tick questing behaviour, non‐human host community composition, seasonal phenology of feeding, and human exposure to ticks (Randolph 2010; Estrada‐Pena *et al.* 2012; Gilbert *et al.* 2014; Ostfeld & Brunner 2015). Despite the variation among different types of vector‐borne diseases, the trait‐based approach applied here is broadly applicable across many diseases (Molnár *et al.* 2017). The nonlinearity of ectotherm trait thermal responses implies that effects of climate change on nearly all infectious diseases will be globally nonlinear but may be locally positive, negative, or neutral depending on host, vector, and parasite trait responses. Crucially, these shifts in disease transmission and burden should be predictable using the type of trait‐based approach presented here (Gehman *et al.* 2018).

In contrast to transmission within suitable environments, current and future geographic range limits on transmission may depend primarily on the capacity of organisms to tolerate heat and cold stress, as well as factors like water availability, land use and vector control (Kearney *et al.* 2009; Overgaard *et al.* 2014; Parham *et al.* 2015). Climate warming may release populations from cold stress near cool range margins (particularly with warming winter temperatures), allowing latitudinal and altitudinal range expansions. By contrast, populations near upper thermal margins will experience increased heat stress that could restrict future geographic ranges (Kingsolver & Woods 2016; Gehman *et al.* 2018). However, many diseases are already much more geographically constrained than their physiological thermal limits (e.g., malaria is now restricted to the tropics but historically occurred throughout temperate and tropical zones), implying that climate change may not expand their range boundaries (Gething *et al.* 2010). Moreover, changes in temperature extremes may have a greater impact on range limits than changes in mean temperatures. Incorporating changes in temperature means and variation is critical for understanding how climate change will impact vector‐borne disease transmission (Savage 2004; Paaijmans *et al.* 2010a; Lambrechts *et al.* 2011; Rohr *et al.* 2011; Blanford *et al.* 2013; Waite *et al.* 2019).

The potential for vectors and pathogens to adapt, via plasticity or evolution, to warming climates is an important empirical and theoretical gap (Table 1) (Thomas & Blanford 2003; Kearney *et al.* 2009; Sternberg & Thomas 2014). Pathogens experience different temperature‐driven selective pressures than vectors. For example, for mosquito‐borne disease transmission to occur at warm temperatures, a minimal requirement is that mosquito lifespan, which has consistently low thermal optima and upper limits, must be long enough to complete the extrinsic incubation period, which decreases dramatically at warm temperatures (Fig. 3). Therefore, as the climate warms, mosquito‐borne disease transmission will require mosquitoes to adapt for longer lifespans at high temperatures, currently the main limitation on transmission near upper thermal limits. Yet mosquitoes could maintain high fitness at high temperatures via rapid oviposition cycles and high reproduction rates despite short lifespans, resulting in diverging selective pressures on mosquitoes and pathogens. Moreover, thermal stress tolerance traits that often determine species range boundaries (Kearney *et al.* 2009; Overgaard *et al.* 2014) do not directly affect transmission and may trade off with other fitness‐ and transmission‐relevant traits. The potential for climate adaptation depends on the amount of standing genetic variation and phenotypic plasticity for survival and other traits at high temperatures, the correlations between these traits and of traits with fitness, the velocity of climate change relative to generation times, and the impact of other selective forces (e.g. insecticide resistance) (Sternberg & Thomas 2014; Lefevre *et al.* 2018; Ohm *et al.* 2018).

While physiological effects of temperature on vectors may be relatively predictable, climate‐driven changes in host population size, movement, behaviour, and immunology are much more idiosyncratic. Human populations may respond to climate change via changes in land use and agricultural practices, housing type and density, water storage practices, seasonal migration, relocation between regions, and demographic shifts (Parham *et al.* 2015; Wesolowski *et al.* 2017), which will inevitably vary geographically. As a result, the realised impact of climate change on disease dynamics will depend on physiological changes in vectors and pathogens as well as on behavioural and demographic changes in host populations (Parham *et al.* 2015; Metcalf *et al.* 2017; Wesolowski *et al.* 2017).

Although direct and indirect effects of temperature on vectors, parasites and hosts have a profound effect on transmission, temperature is only one component of a complex network of causality. Our goal is to predict conditions that favour and disfavor transmission. By analogy, warming oceans tend to increase the intensity of hurricanes, yet warm oceans alone do not generate hurricanes. Likewise, steroid use in baseball players makes hitting home runs more likely, but steroids alone do not cause home runs. In the same way, increasing temperature suitability does not cause disease outbreaks, but it can increase their probability and intensity when other necessary conditions align: it is a threat multiplier (Department of Defense 2014).

Physiological effects of temperature on transmission predict that cool locations and seasons are most vulnerable to warming‐driven increases in vector‐borne disease transmission, while warmer regions may see climate‐driven declines or seasonal shifts in transmission. Urbanisation, land conversion, and other landscape changes may act in concert with climate to drive shifts in the burden of vector‐borne disease from cooler‐adapted diseases like *An. gambiae*‐transmitted malaria to warmer‐adapted diseases like *Ae. aegypti*‐transmitted dengue, chikungunya, and Zika (Mordecai *et al. *in review). Already, in the last two decades malaria has declined in Africa (Bhatt *et al.* 2015) and Latin America (Carter *et al.* 2015), which may be attributable to interactive effects of climate and malaria control programs (Thomson *et al.* 2017), while dengue and other viruses transmitted by warm‐adapted *Ae. aegypti* mosquitoes have risen dramatically (Mitchell 2016; Stanaway *et al.* 2016; PAHO WHO & | Chikungunya | Statistic Data 2016), consistent with the combined effects of climate, urbanisation, and declining vector control.

## Conclusions

Temperature is a fundamental, complex and nonlinear driver of vector‐borne disease transmission. Its physiological effects are consistent across a variety of ectotherm vectors and pathogens because of the underlying constraints of ectotherm physiology. Combining these effects into a unified trait‐based transmission framework can facilitate qualitative predictions for the effects of climate change and comparison across systems. For any vector – pathogen system, a limited range of temperatures permits transmission and intermediate temperatures within this range are optimal. Across systems, thermal limits and optima vary. In a changing climate, the transmission of specific diseases may experience seasonal and geographic shifts that include declines, increases, and minimal effects, which are predictable from trait‐based models. At the same time, because thermal limits and optima vary across vectors and pathogens, the relative suitability for transmission of different diseases is expected to change with the climate. Finally, seasonal and inter–annual variation in temperature, variation in rainfall and weather events, changing land use and urbanisation, and human population changes may mitigate or exacerbate the influence of climate change on vector‐borne disease transmission in many settings. Integrative research that builds on thermal physiology as a backbone and incorporates additional drivers of vector, pathogen, and host ecology is critical for understanding the changing ecology of vector‐borne disease.

## Authorship

EAM conceived of, designed and wrote the manuscript. EAM, LRJ, JRR, SJR, VS, MSS, AMSI and MBT contributed to model parameterisation and validation. EAM, LRJ, MN, JRR, SJR, VS, AMSI and MBT obtained funding for the study. All authors contributed to literature reviews in the studies. All authors revised the manuscript and approved of its final version.

## Supporting information

‐ Click here for additional data file.

## Data Availability

All data used in this paper are either already published elsewhere or included within the tables.

## References

[ele13335-bib-0001] Akey, D.H. , Potter, H.W. & Jone, R.H. (1978). Effects of rearing temperature and larval density on longevity, size, and fecundity in the biting gnat Culicoides variipennis. Ann. Entomol. Soc. Am., 71, 411–418.

[ele13335-bib-0002] Alonso, D. , Bouma, M.J. & Pascual, M. (2011). Epidemic malaria and warmer temperatures in recent decades in an East African highland. Proc. R. Soc. B Biol. Sci., 278, 1661–1669. 10.1098/rspb.2010.2020 PMC3081772 21068045

[ele13335-bib-0003] Alsan, M. (2015). The effect of the TseTse fly on African development. Am. Econ. Rev., 105, 382–410.

[ele13335-bib-0004] Amarasekare, P. & Savage, V. (2012). A framework for elucidating the temperature dependence of fitness. Am. Nat., 179, 178–191. 22218308 10.1086/663677

[ele13335-bib-0005] Angilletta, M.J. (2009). Thermal Adaptation: A Theoretical and Empirical Synthesis. Oxford, UK:Oxford University Press.

[ele13335-bib-0006] Bacaër, N. & Ait Dads, E.H. (2012). On the biological interpretation of a definition for the parameter R 0 in periodic population models. J. Math. Biol., 65, 601–621. 21987087 10.1007/s00285-011-0479-4

[ele13335-bib-0007] Bacaër, N. & Guernaoui, S. (2006). The epidemic threshold of vector‐borne diseases with seasonality. J. Math. Biol., 53, 421–436. 16823580 10.1007/s00285-006-0015-0

[ele13335-bib-0008] Bayoh, M.N. (2001). Studies on the development and survival of anopheles gambiae sensu stricto at various temperatures and relative humidities. Durham University, Doctoral.

[ele13335-bib-0009] Bernhardt, J.R. , Sunday, J.M. , Thompson, P.L. & O’Connor, M.I. (2018). Nonlinear averaging of thermal experience predicts population growth rates in a thermally variable environment. Proc R Soc B, 285, 20181076. 10.1098/rspb.2018.1076 PMC6158538 30209223

[ele13335-bib-0010] Bhatt, S. , Weiss, D.J. , Cameron, E. , Bisanzio, D. , Mappin, B. , Dalrymple, U. , et al. (2015). The effect of malaria control on Plasmodium falciparum in Africa between 2000 and 2015. Nature, 526, 207–211. 26375008 10.1038/nature15535 PMC4820050

[ele13335-bib-0011] Bi, P. , Hiller, J.E. , Cameron, A.S. , Zhang, Y. & Givney, R. (2009). Climate variability and Ross River virus infections in Riverland, South Australia, 1992–2004. Epidemiol. Infect., 137, 1486–1493. 19296873 10.1017/S0950268809002441

[ele13335-bib-0012] Blanford, J.I. , Blanford, S. , Crane, R.G. , Mann, M.E. , Paaijmans, K.P. , Schreiber, K.V. , et al. (2013). Implications of temperature variation for malaria parasite development across Africa. Sci. Rep., 3, 1300. 23419595 10.1038/srep01300 PMC3575117

[ele13335-bib-0013] Brady, O.J. , Golding, N. , Pigott, D.M. , Kraemer, M.U.G. , Messina, J.P. , Reiner, R.C. Jr. , et al. (2014). Global temperature constraints on Aedes aegypti and Ae. albopictus persistence and competence for dengue virus transmission. Parasit. Vectors, 7, 338. 25052008 10.1186/1756-3305-7-338 PMC4148136

[ele13335-bib-0014] Briere, J.‐F. , Pracros, P. , Le Roux, A.‐Y. & Pierre, J.‐S. (1999). A novel rate model of temperature‐dependent development for arthropods. Environ. Entomol., 28, 22–29.

[ele13335-bib-0015] Brown, J.H. , Gillooly, J.F. , Allen, A.P. , Savage, V.M. & West, G.B. (2004). Toward a metabolic theory of ecology. Ecology, 85, 1771–1789.

[ele13335-bib-0016] Buckley, L.B. , Urban, M.C. , Angilletta, M.J. , Crozier, L.G. , Rissler, L.J. & Sears, M.W. (2010). Can mechanism inform species’ distribution models? Ecol. Lett., 13, 1041–1054. 20482574 10.1111/j.1461-0248.2010.01479.x

[ele13335-bib-0017] Caminade, C. , Kovats, S. , Rocklov, J. , Tompkins, A.M. , Morse, A.P. , Colón‐González, F.J. , et al. (2014). Impact of climate change on global malaria distribution. Proc. Natl Acad. Sci., 111, 3286–3291. 24596427 10.1073/pnas.1302089111 PMC3948226

[ele13335-bib-0018] Caminade, C. , Turner, J. , Metelmann, S. , Hesson, J.C. , Blagrove, M.S.C. , Solomon, T. , et al. (2017). Global risk model for vector‐borne transmission of Zika virus reveals the role of El Niño 2015. Proc. Natl Acad. Sci., 114, 119–124. 27994145 10.1073/pnas.1614303114 PMC5224381

[ele13335-bib-0019] Carlson, C.J. , Dougherty, E. , Boots, M. , Getz, W. & Ryan, S.J. (2018). Consensus and conflict among ecological forecasts of Zika virus outbreaks in the United States. Sci. Rep., 8, 4921. 29563545 10.1038/s41598-018-22989-0 PMC5862882

[ele13335-bib-0020] Carpenter, S. , Wilson, A. , Barber, J. , Veronesi, E. , Mellor, P. , Venter, G. , et al. (2011). Temperature dependence of the extrinsic incubation period of orbiviruses in Culicoides biting midges. PLoS ONE, 6, e27987. 22125649 10.1371/journal.pone.0027987 PMC3220716

[ele13335-bib-0021] Carter, K.H. , Singh, P. , Mujica, O.J. , Escalada, R.P. , Ade, M.P. , Castellanos, L.G. , et al. (2015). Malaria in the Americas: trends from 1959 to 2011. Am. J. Trop. Med. Hyg., 92, 302–316. 25548378 10.4269/ajtmh.14-0368 PMC4347333

[ele13335-bib-0022] Cator, L. , Johnson, L.R. , Mordecai, E.A. , Moustaid, F.E. , Smallwood, T. , Deau, S.L. , et al. (2019). More than a flying syringe: using functional traits in vector borne disease research. bioRxiv, 501320.

[ele13335-bib-0023] Cheng, A. , Chen, D. , Woodstock, K. , Ogden, N.H. , Wu, X. & Wu, J. (2017). Analyzing the potential risk of climate change on lyme disease in eastern ontario, Canada using time series remotely sensed temperature data and tick population modelling. Remote Sens., 9, 609.

[ele13335-bib-0024] Cohen, J.M. , Civitello, D.J. , Brace, A.J. , Feichtinger, E.M. , Ortega, C.N. , Richardson, J.C. , et al. (2016). Spatial scale modulates the strength of ecological processes driving disease distributions. Proc. Natl Acad. Sci., 113, E3359–E3364. 201521657 27247398 10.1073/pnas.1521657113 PMC4914148

[ele13335-bib-0025] Cohen, J.M. , Civitello, D.J. , Venesky, M.D. , McMahon, T.A. & Rohr, J.R. (2019). An interaction between climate change and infectious disease drove widespread amphibian declines. Glob. Change Biol., 25, 927–937. 10.1111/gcb.14489 30484936

[ele13335-bib-0026] Cox, F.E. (2010). History of the discovery of the malaria parasites and their vectors. Parasit. Vectors, 3, 5. 20205846 10.1186/1756-3305-3-5 PMC2825508

[ele13335-bib-0027] Craig, M.H. , Snow, R.W. & le Sueur, D. (1999). A climate‐based distribution model of malaria transmission in sub‐saharan Africa. Parasitol. Today, 15, 105–111. 10322323 10.1016/s0169-4758(99)01396-4

[ele13335-bib-0028] Dell, A.I. , Pawar, S. & Savage, V.M. (2011). Systematic variation in the temperature dependence of physiological and ecological traits. Proc. Natl. Acad. Sci., 108, 10591–10596. 21606358 10.1073/pnas.1015178108 PMC3127911

[ele13335-bib-0029] Department of Defense (2014). Quadrennial Defense Review 2014. United States Department of Defense, Washington D.C.

[ele13335-bib-0030] Deutsch, C.A. , Tewksbury, J.J. , Huey, R.B. , Sheldon, K.S. , Ghalambor, C.K. , Haak, D.C. , et al. (2008). Impacts of climate warming on terrestrial ectotherms across latitude. Proc. Natl Acad. Sci., 105, 6668–6672. 18458348 10.1073/pnas.0709472105 PMC2373333

[ele13335-bib-0031] Diekmann, O. , Heesterbeek, J.A.P. & Roberts, M.G. (2009). The construction of next‐generation matrices for compartmental epidemic models. J. R. Soc. Interface, 7, 873–885. 19892718 10.1098/rsif.2009.0386 PMC2871801

[ele13335-bib-0032] Dietz, K. (1993). The estimation of the basic reproduction number for infectious diseases. Stat. Methods Med. Res., 2, 23–41. 8261248 10.1177/096228029300200103

[ele13335-bib-0033] Duffy, M.R. , Chen, T.‐H. , Hancock, W.T. , Powers, A.M. , Kool, J.L. , Lanciotti, R.S. , et al. (2009). Zika virus outbreak on yap Island, federated states of micronesia. N. Engl. J. Med., 360, 2536–2543. 19516034 10.1056/NEJMoa0805715

[ele13335-bib-0034] Ermert, V. , Fink, A.H. , Morse, A.P. & Paeth, H. (2012). The impact of regional climate change on malaria risk due to greenhouse forcing and land‐use changes in tropical Africa. Environ. Health Perspect., 120, 77–84. 21900078 10.1289/ehp.1103681 PMC3261943

[ele13335-bib-0035] Escobar, L.E. , Romero‐Alvarez, D. , Leon, R. , Lepe‐Lopez, M.A. , Craft, M.E. , Borbor‐Cordova, M.J. , et al. (2016). Declining Prevalence of Disease Vectors Under Climate Change. Sci. Rep., 6, 39150. 27982119 10.1038/srep39150 PMC5159793

[ele13335-bib-0036] Estrada-Peña, A. , Ayllon, N. & De La Fuente, J. (2012). Impact of climate trends on tick‐borne pathogen transmission. Front. Physiol., 3, 64. 22470348 10.3389/fphys.2012.00064 PMC3313475

[ele13335-bib-0037] Fros, J.J. , Geertsema, C. , Vogels, C.B. , Roosjen, P.P. , Failloux, A.‐B. , Vlak, J.M. , et al. (2015). West nile virus: High transmission rate in North‐Western European mosquitoes indicates Its epidemic potential and warrants increased surveillance. PLoS Negl. Trop. Dis., 9, e0003956. 26225555 10.1371/journal.pntd.0003956 PMC4520649

[ele13335-bib-0038] Gatton, M.L. , Kay, B.H. & Ryan, P.A. (2005). Environmental predictors of Ross River virus disease outbreaks in Queensland. Australia. Am. J. Trop. Med. Hyg., 72, 792–799. 15964965

[ele13335-bib-0039] Gehman, A.‐L.M. , Hall, R.J. & Byers, J.E. (2018). Host and parasite thermal ecology jointly determine the effect of climate warming on epidemic dynamics. Proc. Natl Acad. Sci, 115, 744–749. 29311324 10.1073/pnas.1705067115 PMC5789902

[ele13335-bib-0040] Gething, P.W. , Smith, D.L. , Patil, A.P. , Tatem, A.J. , Snow, R.W. & Hay, S.I. (2010). Climate change and the global malaria recession. Nature, 465, 342–345. 20485434 10.1038/nature09098 PMC2885436

[ele13335-bib-0041] Gilbert, L. , Aungier, J. & Tomkins, J.L. (2014). Climate of origin affects tick (Ixodes ricinus) host‐seeking behavior in response to temperature: implications for resilience to climate change? Ecol. Evol., 4, 1186–1198. 24772293 10.1002/ece3.1014 PMC3997332

[ele13335-bib-0042] Hay, S.I. , Rogers, D.J. , Toomer, J.F. & Snow, R.W. (2000). Annual Plasmodium falciparum entomological inoculation rates (EIR) across Africa: literature survey, internet access and review. Trans. R. Soc. Trop. Med. Hyg., 94, 113–127. 10897348 10.1016/s0035-9203(00)90246-3 PMC3204456

[ele13335-bib-0043] Hechinger, R.F. (2013). A metabolic and body‐size scaling framework for parasite within‐host abundance, biomass, and energy flux. Am. Nat., 182, 234–248. 23852357 10.1086/670820

[ele13335-bib-0044] Heesterbeek, J.A.P, (2002). A brief history of R0 and a recipe for its calculation. Acta Biotheor., 50, 189–204. 12211331 10.1023/a:1016599411804

[ele13335-bib-0045] Heffernan, J.M. , Smith, R.J. & Wahl, L.M. (2005). Perspectives on the basic reproductive ratio. J. R. Soc. Interface, 2, 281–293. 16849186 10.1098/rsif.2005.0042 PMC1578275

[ele13335-bib-0046] Helmuth, B. , Kingsolver, J.G. & Carrington, E. (2005). Biophysics, physiological ecology, and climate change: does Mechanism Matter? Annu. Rev. Physiol., 67, 177–201. 15709956 10.1146/annurev.physiol.67.040403.105027

[ele13335-bib-0047] Hooten, M.B. & Hobbs, N.T. (2015). A guide to Bayesian model selection for ecologists. Ecol. Monogr., 85, 3–28.

[ele13335-bib-0048] Hu, W. , Clements, A. , Williams, G. , Tong, S. & Mengersen, K. (2010). Bayesian spatiotemporal analysis of socio‐ecologic drivers of ross River virus transmission in queensland. Australia. Am. J. Trop. Med. Hyg., 83, 722–728. 20810846 10.4269/ajtmh.2010.09-0551 PMC2929077

[ele13335-bib-0049] Huber, J.H. , Johnston, G.L. , Greenhouse, B. , Smith, D.L. & Perkins, T.A. (2016). Quantitative, model‐based estimates of variability in the generation and serial intervals of Plasmodium falciparum malaria. Malar. J., 15, 490. 27660051 10.1186/s12936-016-1537-6 PMC5034682

[ele13335-bib-0050] Huber, J.H. , Childs, M.L. , Caldwell, J.M. & Mordecai, E.A. (2018). Seasonal temperature variation influences climate suitability for dengue, chikungunya, and Zika transmission. PLoS Negl. Trop. Dis., 12, e0006451. 29746468 10.1371/journal.pntd.0006451 PMC5963813

[ele13335-bib-0051] Huey, R.B. & Berrigan, D. (2001). Temperature, demography, and ectotherm fitness. Am. Nat., 158, 204–210. 18707349 10.1086/321314

[ele13335-bib-0052] Huey, R.B. & Kingsolver, J.G. (2011). Variation in universal temperature dependence of biological rates. Proc. Natl Acad. Sci., 108, 10377–10378. 21680885 10.1073/pnas.1107430108 PMC3127921

[ele13335-bib-0053] Jaramillo‐Ochoa, R. , Sippy, R. , Farrell, D.F. , Cueva‐Aponte, C. , Beltrán‐Ayala, E. , Gonzaga, J.L. , et al. (2019). Effects of political instability in Venezuela on malaria resurgence at ecuador‐peru border, 2018. Emerg. Infect. Dis., 25, 834–836. 30698522 10.3201/eid2504.181355 PMC6433012

[ele13335-bib-0054] Johansson, M.A. , Powers, A.M. , Pesik, N. , Cohen, N.J. & Staples, J.E. (2014). Nowcasting the spread of chikungunya virus in the Americas. PLoS ONE, 9, e104915. 25111394 10.1371/journal.pone.0104915 PMC4128737

[ele13335-bib-0055] Johnson, F.H. & Lewin, I. (1946). The growth rate of E. coli in relation to temperature, quinine and coenzyme. J. Cell. Comp. Physiol., 28, 47–75. 21002957 10.1002/jcp.1030280104

[ele13335-bib-0056] Johnson, L.R. , Ben‐Horin, T. , Lafferty, K.D. , McNally, A. , Mordecai, E. , Paaijmans, K.P. , et al. (2015). Understanding uncertainty in temperature effects on vector‐borne disease: a Bayesian approach. Ecology, 96, 203–213. 26236905 10.1890/13-1964.1

[ele13335-bib-0057] Kearney, M. & Porter, W. (2009). Mechanistic niche modelling: combining physiological and spatial data to predict species’ ranges. Ecol. Lett., 12, 334–350. 19292794 10.1111/j.1461-0248.2008.01277.x

[ele13335-bib-0058] Kearney, M. , Porter, W.P. , Williams, C. , Ritchie, S. & Hoffmann, A.A. (2009). Integrating biophysical models and evolutionary theory to predict climatic impacts on species’ ranges: the dengue mosquito Aedes aegypti in Australia. Funct. Ecol., 23, 528–538.

[ele13335-bib-0059] Kearney, M. , Simpson, S.J. , Raubenheimer, D. & Helmuth, B. (2010). Modelling the ecological niche from functional traits. Philos. Trans. R. Soc. B Biol. Sci., 365, 3469–3483. 10.1098/rstb.2010.0034 PMC2981966 20921046

[ele13335-bib-0060] Kingsolver, J.G. (2009). The well‐temperatured biologist: (American Society of Naturalists Presidential Address). Am. Nat., 174, 755–768. 19857158 10.1086/648310

[ele13335-bib-0061] Kingsolver, J.G. & Woods, H.A. (2016). Beyond thermal performance curves: Modeling time‐dependent effects of thermal stress on ectotherm growth rates. Am. Nat., 187, 283–294. 26913942 10.1086/684786

[ele13335-bib-0062] Kirk, D. , Jones, N. , Peacock, S. , Phillips, J. , Molnár, P.K. , Krkošek, M. , et al. (2018). Empirical evidence that metabolic theory describes the temperature dependency of within‐host parasite dynamics. PLOS Biol., 16, e2004608. 29415043 10.1371/journal.pbio.2004608 PMC5819823

[ele13335-bib-0063] Kirk, D. , Luijckx, P. , Stanic, A. & Krkošek, M. (2019). Predicting the thermal and allometric dependencies of disease transmission via the metabolic theory of ecology. Am. Nat., 193, 661–676. 31002572 10.1086/702846

[ele13335-bib-0064] Koolhof, I.S. , Bettiol, S. & Carver, S. (2017). Fine‐temporal forecasting of outbreak probability and severity: Ross River virus in Western Australia. Epidemiol. Infect., 145, 2949–2960. 28868994 10.1017/S095026881700190X PMC9152751

[ele13335-bib-0065] Kramer, A.M. , Pulliam, J.T. , Alexander, L.W. , Park, A.W. , Rohani, P. & Drake, J.M. (2016). Spatial spread of the West Africa Ebola epidemic. R. Soc. Open Sci., 3, 160294. 27853607 10.1098/rsos.160294 PMC5108957

[ele13335-bib-0066] Krisher, L.K. , Krisher, J. , Ambuludi, M. , Arichabala, A. , Beltrán‐Ayala, E. , Navarrete, P. , et al. (2016). Successful malaria elimination in the Ecuador‐Peru border region: epidemiology and lessons learned. Malar. J., 15, 573. 27894320 10.1186/s12936-016-1630-x PMC5126842

[ele13335-bib-0067] Kushmaro, A. , Awerbuch‐Friedlander, T. & Levins, R. (2015). Temperature effects on the basic reporudctive number (R0) of West Nile virus, based on ecological parameters: Endemic vs. new emergence regions. J. Trop., Dis, S1–001.

[ele13335-bib-0068] Lafferty, K.D. (2009). The ecology of climate change and infectious diseases. Ecology, 90, 888–900. 19449681 10.1890/08-0079.1

[ele13335-bib-0069] Lambrechts, L. , Paaijmans, K.P. , Fansiri, T. , Carrington, L.B. , Kramer, L.D. , Thomas, M.B. , et al. (2011). Impact of daily temperature fluctuations on dengue virus transmission by Aedes aegypti. Proc. Natl Acad. Sci., 108, 7460–7465. 21502510 10.1073/pnas.1101377108 PMC3088608

[ele13335-bib-0070] Lefevre, T. , Ohm, J. , Dabiré, K.R. , Cohuet, A. , Choisy, M. , Thomas, M.B. , et al. (2018). Transmission traits of malaria parasites within the mosquito: Genetic variation, phenotypic plasticity, and consequences for control. Evol. Appl., 11, 456–469. 29636799 10.1111/eva.12571 PMC5891056

[ele13335-bib-0071] Li, R. , Xu, L. , Bjørnstad, O.N. , Liu, K. , Song, T. , Chen, A. , et al. (2019). Climate‐driven variation in mosquito density predicts the spatiotemporal dynamics of dengue. Proc. Natl Acad. Sci., 116, 3624–3629. 30808752 10.1073/pnas.1806094116 PMC6397594

[ele13335-bib-0072] Little, E. , Bajwa, W. & Shaman, J. (2017). Local environmental and meteorological conditions influencing the invasive mosquito Ae. albopictus and arbovirus transmission risk in New York City. PLoS Negl. Trop. Dis., 11, e0005828. 28832586 10.1371/journal.pntd.0005828 PMC5584979

[ele13335-bib-0073] Liu‐Helmersson, J. , Stenlund, H. , Wilder‐Smith, A. & Rocklöv, J. (2014). Vectorial capacity of Aedes aegypti: effects of temperature and implications for global dengue epidemic potential. PLoS ONE, 9, e89783. 24603439 10.1371/journal.pone.0089783 PMC3946027

[ele13335-bib-0074] Loarie, S.R. , Duffy, P.B. , Hamilton, H. , Asner, G.P. , Field, C.B. & Ackerly, D.D. (2009). The velocity of climate change. Nature, 462, 1052–1055. 20033047 10.1038/nature08649

[ele13335-bib-0075] Lowe, R. , Gasparrini, A. , Meerbeeck, C.J.V. , Lippi, C.A. , Mahon, R. , Trotman, A.R. , et al. (2018). Nonlinear and delayed impacts of climate on dengue risk in Barbados: A modelling study. PLoS Med., 15, e1002613. 30016319 10.1371/journal.pmed.1002613 PMC6049902

[ele13335-bib-0076] Martens, W.J.M. , Jetten, T.H. & Focks, D.A. (1997). Sensitivity of malaria, schistosomiasis and dengue to global warming. Clim. Change, 35, 145–156.

[ele13335-bib-0077] Martin, T.L. & Huey, R.B. (2008). Why “Suboptimal” Is optimal: Jensen’s inequality and ectotherm thermal preferences. Am. Nat., 171, E102–E118. 18271721 10.1086/527502

[ele13335-bib-0078] Mena, N. , Troyo, A. , Bonilla‐Carrión, R. & Calderón‐Arguedas, Ó. (2011). Factores asociados con la incidencia de dengue en Costa Rica. Rev. Panam. Salud Pública, 29, 234–242. 21603768 10.1590/s1020-49892011000400004

[ele13335-bib-0079] Metcalf, C.J.E. , Walter, K.S. , Wesolowski, A. , Buckee, C.O. , Shevliakova, E. , Tatem, A.J. , et al. (2017). Identifying climate drivers of infectious disease dynamics: recent advances and challenges ahead. Proc R Soc B, 284, 20170901. 10.1098/rspb.2017.0901 PMC5563806 28814655

[ele13335-bib-0080] Mitchell, C. (2016). PAHO WHO | PAHO Statement on Zika Virus Transmission and Prevention. Pan Am. Health Organ. World Health Organ. Available at: http://www.paho.org/hq/index.php?option=com_content&view=article&xml:id=11605&Itemxml:id=41716&xml:lang=en . Last accessed 5 May 2016.

[ele13335-bib-0081] Molnár, P.K. , Kutz, S.J. , Hoar, B.M. & Dobson, A.P. (2013). Metabolic approaches to understanding climate change impacts on seasonal host‐macroparasite dynamics. Ecol. Lett., 16, 9–21. 23157563 10.1111/ele.12022

[ele13335-bib-0082] Molnár, P.K. , Sckrabulis, J.P. , Altman, K.A. & Raffel, T.R. (2017). Thermal performance curves and the metabolic theory of ecology—a practical guide to models and experiments for parasitologists. J. Parasitol., 103, 423–439. 28604284 10.1645/16-148

[ele13335-bib-0083] Moore, S. , Shrestha, S. , Tomlinson, K.W. & Vuong, H. (2012). Predicting the effect of climate change on African trypanosomiasis: integrating epidemiology with parasite and vector biology. J. R. Soc. Interface, 9, 817–830. 22072451 10.1098/rsif.2011.0654 PMC3306657

[ele13335-bib-0084] Mordecai, E.A. , Ryan, S.J. , Caldwell, J.M. , Shah, M. & LaBeaud, A.D. (in review). Climate change could shift disease burden from malaria to arboviruses in Africa. Nat. Clim. Change. 10.1016/S2542-5196(20)30178-9 PMC7490804 32918887

[ele13335-bib-0085] Mordecai, E.A. , Paaijmans, K.P. , Johnson, L.R. , Balzer, C.H. , Ben‐Horin, T. , de Moor, E. , et al. (2013). Optimal temperature for malaria transmission is dramatically lower than previously predicted. Ecol. Lett., 16, 22–30. 23050931 10.1111/ele.12015

[ele13335-bib-0086] Mordecai, E.A. , Cohen, J.M. , Evans, M.V. , Gudapati, P. , Johnson, L.R. , Lippi, C.A. , et al. (2017). Detecting the impact of temperature on transmission of Zika, dengue, and chikungunya using mechanistic models. PLoS Negl. Trop. Dis., 11, e0005568. 28448507 10.1371/journal.pntd.0005568 PMC5423694

[ele13335-bib-0087] Morin, C.W. , Monaghan, A.J. , Hayden, M.H. , Barrera, R. & Ernst, K. (2015). Meteorologically driven simulations of dengue epidemics in San Juan. PR. PLoS Negl Trop Dis, 9, e0004002. 26275146 10.1371/journal.pntd.0004002 PMC4537107

[ele13335-bib-0088] Murdock, C.C. , Blanford, S. , Luckhart, S. & Thomas, M.B. (2014). Ambient temperature and dietary supplementation interact to shape mosquito vector competence for malaria. J. Insect Physiol., 67, 37–44. 24911425 10.1016/j.jinsphys.2014.05.020 PMC4107084

[ele13335-bib-0089] Murdock, C.C. , Evans, M.V. , McClanahan, T.D. , Miazgowicz, K.L. & Tesla, B. (2017). Fine‐scale variation in microclimate across an urban landscape shapes variation in mosquito population dynamics and the potential of Aedes albopictus to transmit arboviral disease. PLoS Negl. Trop. Dis., 11, e0005640. 28558030 10.1371/journal.pntd.0005640 PMC5466343

[ele13335-bib-0090] O’Connor, M.I. & Bernhardt, J.R. (2018). The metabolic theory of ecology and the cost of parasitism. PLOS Biol., 16, e2005628. 29608559 10.1371/journal.pbio.2005628 PMC5897036

[ele13335-bib-0091] Ohm, J.R. , Baldini, F. , Barreaux, P. , Lefevre, T. , Lynch, P.A. , Suh, E. , et al. (2018). Rethinking the extrinsic incubation period of malaria parasites. Parasit. Vectors, 11, 178. 29530073 10.1186/s13071-018-2761-4 PMC5848458

[ele13335-bib-0092] Ostfeld, R.S. & Brunner, J.L. (2015). Climate change and Ixodes tick‐borne diseases of humans. Philos. Trans. R. Soc. B Biol. Sci., 370, 20140051. 10.1098/rstb.2014.0051 PMC4342967 25688022

[ele13335-bib-0093] Overgaard, J. , Kearney, M.R. & Hoffmann, A.A. (2014). Sensitivity to thermal extremes in Australian Drosophila implies similar impacts of climate change on the distribution of widespread and tropical species. Glob. Change Biol., 20, 1738–1750. 10.1111/gcb.12521 24549716

[ele13335-bib-0094] Paaijmans, K.P. & Thomas, M.B. (2011a). Health: Wealth versus warming. Nat. Clim. Change, 1, 349–350.

[ele13335-bib-0095] Paaijmans, K.P. & Thomas, M.B. (2011b). The influence of mosquito resting behaviour and associated microclimate for malaria risk. Malar. J., 10, 183. 21736735 10.1186/1475-2875-10-183 PMC3146900

[ele13335-bib-0096] Paaijmans, K.P. , Blanford, S. , Bell, A.S. , Blanford, J.I. , Read, A.F. & Thomas, M.B. (2010a). Influence of climate on malaria transmission depends on daily temperature variation. Proc. Natl Acad. Sci., 107, 15135–15139. 20696913 10.1073/pnas.1006422107 PMC2930540

[ele13335-bib-0097] Paaijmans, K.P. , Imbahale, S.S. , Thomas, M.B. & Takken, W. (2010b). Relevant microclimate for determining the development rate of malaria mosquitoes and possible implications of climate change. Malar. J., 9, 196. 20618930 10.1186/1475-2875-9-196 PMC2912924

[ele13335-bib-0098] Paaijmans, K.P. , Blanford, S. , Chan, B.H.K. & Thomas, M.B. (2011). Warmer temperatures reduce the vectorial capacity of malaria mosquitoes. Biol. Lett., 8, 465–468, rsbl20111075. 22188673 10.1098/rsbl.2011.1075 PMC3367745

[ele13335-bib-0099] Paaijmans, K.P. , Heinig, R.L. , Seliga, R.A. , Blanford, J.I. , Blanford, S. , Murdock, C.C. , et al. (2013). Temperature variation makes ectotherms more sensitive to climate change. Glob. Change Biol., 19, 2373–2380. 10.1111/gcb.12240 PMC3908367 23630036

[ele13335-bib-0100] PAHO WHO & | Chikungunya | Statistic Data . (2016). Available at: http://www.paho.org/hq/index.php?option=com_topics&view=readall&cxml:id=5927&Itemxml:id=40931&xml:lang=en Last accessed 10 July 2016 .

[ele13335-bib-0101] Parham, P. & Michael, E. (2010). Modeling the effects of weather and climate change on malaria transmission. Environ. Health Perspect., 118, 620–626. 20435552 10.1289/ehp.0901256 PMC2866676

[ele13335-bib-0102] Parham, P.E. , Waldock, J. , Christophides, G.K. , Hemming, D. , Agusto, F. , Evans, K.J. , et al. (2015). Climate, environmental and socio‐economic change: weighing up the balance in vector‐borne disease transmission. Biol. Sci., 370, 20130551–20130551. 10.1098/rstb.2013.0551 PMC4342957 25688012

[ele13335-bib-0103] Pascual, M. & Bouma, M.J. (2009). Do rising temperatures matter? Ecology, 90, 906–912. 19449684 10.1890/08-0730.1

[ele13335-bib-0104] Pascual, M. , Ahumada, J. , Chaves, L. , Rodo, X. & Bouma, M. (2006). Malaria resurgence in the East African highlands: temperature trends revisited. Proc. Natl Acad. Sci., 103, 5829–5834. 16571662 10.1073/pnas.0508929103 PMC1416896

[ele13335-bib-0105] Paull, S.H. , Horton, D.E. , Ashfaq, M. , Rastogi, D. , Kramer, L.D. , Diffenbaugh, N.S. , et al. (2017). Drought and immunity determine the intensity of West Nile virus epidemics and climate change impacts. Proc. R. Soc. B Biol. Sci., 284, 20162078. 10.1098/rspb.2016.2078 PMC5310598 28179512

[ele13335-bib-0106] Peña‐García, V. , Triana‐Chávez, O. , Mejía‐Jaramillo, A. , Díaz, F. , Gómez‐Palacio, A. , Arboleda‐Sánchez, S. , et al. (2016). Infection rates by dengue virus in mosquitoes and the influence of temperature may be related to different endemicity patterns in three colombian cities. Int. J. Environ. Res. Public. Health, 13, 734. 10.3390/ijerph13070734 PMC4962275 27455289

[ele13335-bib-0107] Peña‐García, V.H. , Triana‐Chávez, O. & Arboleda‐Sánchez, S. (2017). Estimating effects of temperature on dengue transmission in colombian cities. Ann. Glob., Health, 83, 509–518. 29221523 10.1016/j.aogh.2017.10.011

[ele13335-bib-0108] Perkins, T.A. , Metcalf, C.J.E. , Grenfell, B.T. & Tatem, A.J. (2015). Estimating drivers of autochthonous transmission of chikungunya virus in its invasion of the Americas. PLoS Curr., 7. 10.1371/currents.outbreaks.a4c7b6ac10e0420b1788c9767946d1fc PMC4339250 25737803

[ele13335-bib-0109] Perkins, T.A. , Siraj, A.S. , Ruktanonchai, C.W. , Kraemer, M.U.G. & Tatem, A.J. (2016). Model‐based projections of Zika virus infections in childbearing women in the Americas. Nat. Microbiol., 1, 16126. 27562260 10.1038/nmicrobiol.2016.126

[ele13335-bib-0110] Qiao, H. , Peterson, A.T. , Campbell, L.P. , Soberón, J. , Ji, L. & Escobar, L.E. (2016). NicheA: creating virtual species and ecological niches in multivariate environmental scenarios. Ecography, 39, 805–813.

[ele13335-bib-0111] Randolph, S.E. (2010). To what extent has climate change contributed to the recent epidemiology of tick‐borne diseases? *Vet. Parasitol* . Ticks and Tick‐borne Pathogens, 167, 92–94. 10.1016/j.vetpar.2009.09.011 19833440

[ele13335-bib-0112] Reiner, R.C. , Perkins, T.A. , Barker, C.M. , Niu, T. , Chaves, L.F. , Ellis, A.M. , et al. (2013). A systematic review of mathematical models of mosquito‐borne pathogen transmission: 1970–2010. J. R. Soc. Interface, 10, 20120921. 23407571 10.1098/rsif.2012.0921 PMC3627099

[ele13335-bib-0113] Ren, Z. , Wang, D. , Ma, A. , Hwang, J. , Bennett, A. , Sturrock, H.J.W. , et al. (2016). Predicting malaria vector distribution under climate change scenarios in China: Challenges for malaria elimination. Sci. Rep., 6, 20604. 26868185 10.1038/srep20604 PMC4751525

[ele13335-bib-0114] Rodriguez‐Barraquer, I. , Cordeiro, M.T. , Braga, C. , de Souza, W.V. , Marques, E.T. & Cummings, D.A.T. (2011). From re‐emergence to hyperendemicity: The natural history of the dengue epidemic in Brazil. PLoS Negl. Trop. Dis., 5, e935. 21245922 10.1371/journal.pntd.0000935 PMC3014978

[ele13335-bib-0115] Rogers, D.J. & Randolph, S.E. (2006). Climate change and vector‐borne diseases. Adv. Parasitol., 62, 345–381. 16647975 10.1016/S0065-308X(05)62010-6

[ele13335-bib-0116] Rohr, J.R. , Dobson, A.P. , Johnson, P.T.J. , Kilpatrick, A.M. , Paull, S.H. , Raffel, T.R. , et al. (2011). Frontiers in climate change–disease research. Trends Ecol. Evol., 26, 270–277. 21481487 10.1016/j.tree.2011.03.002 PMC3374867

[ele13335-bib-0117] Rohr, J.R. , Civitello, D.J. , Cohen, J.M. , Roznik, E.A. , Sinervo, B. & Dell, A.I. (2018). The complex drivers of thermal acclimation and breadth in ectotherms. Ecol. Lett., 21, 1425–1439. 30009486 10.1111/ele.13107

[ele13335-bib-0118] Ruybal, J.E. , Kramer, L.D. & Kilpatrick, A.M. (2016). Geographic variation in the response of Culex pipiens life history traits to temperature. Parasit. Vectors, 9, 116. 26928181 10.1186/s13071-016-1402-z PMC4772444

[ele13335-bib-0119] Ryan, S.J. , McNally, A. , Johnson, L.R. , Mordecai, E.A. , Ben‐Horin, T. , Paaijmans, K. , et al. (2015). Mapping physiological suitability limits for malaria in Africa under climate change. Vector‐Borne Zoonotic Dis., 15, 718–725. 26579951 10.1089/vbz.2015.1822 PMC4700390

[ele13335-bib-0120] Ryan, S.J. , Carlson, C.J. , Mordecai, E.A. & Johnson, L.R. (2019). Global expansion and redistribution of Aedes‐borne virus transmission risk with climate change. PLoS Negl. Trop. Dis., 13, e0007213. 30921321 10.1371/journal.pntd.0007213 PMC6438455

[ele13335-bib-0121] Salje, H. , Lessler, J. , Berry, I.M. , Melendrez, M.C. , Endy, T. , Kalayanarooj, S. , et al. (2017). Dengue diversity across spatial and temporal scales: Local structure and the effect of host population size. Science, 355, 1302–1306. 28336667 10.1126/science.aaj9384 PMC5777672

[ele13335-bib-0122] Salje, H. , Cummings, D.A.T. , Rodriguez‐Barraquer, I. , Katzelnick, L.C. , Lessler, J. , Klungthong, C. , et al. (2018). Reconstruction of antibody dynamics and infection histories to evaluate dengue risk. Nature, 557, 719–723. 29795354 10.1038/s41586-018-0157-4 PMC6064976

[ele13335-bib-0123] Santos‐Vega, M. , Lowe, R. , Anselin, L. , Desai, V. , Vaishnav, K.G. , Naik, A. , et al. (2019). Local climate and social inequality drive spatio‐temporal variation in malaria risk across a complex urban landscape. bioRxiv, 583880 10.1101/583880

[ele13335-bib-0124] Savage, V.M. (2004). Improved approximations to scaling relationships for species, populations, and ecosystems across latitudinal and elevational gradients. J. Theor. Biol., 227, 525–534. 15038987 10.1016/j.jtbi.2003.11.030

[ele13335-bib-0125] Shah, M. , Krystosik, A.R. , Ndenga, B.A. , Mutuku, F.M. , Caldwell, J.M. , Otuka, V. , et al. (2019). Malaria smear positivity among Kenyan children peaks at intermediate temperatures as predicted by ecological models. Parasit. Vectors, 12, 288. 31171037 10.1186/s13071-019-3547-z PMC6555721

[ele13335-bib-0126] Shapiro, L.L.M. , Whitehead, S.A. & Thomas, M.B. (2017). Quantifying the effects of temperature on mosquito and parasite traits that determine the transmission potential of human malaria. PLOS Biol., 15, e2003489. 29036170 10.1371/journal.pbio.2003489 PMC5658182

[ele13335-bib-0127] Shocket, M.S. , Ryan, S.J. & Mordecai, E.A. (2018). Temperature explains broad patterns of Ross River virus transmission. eLife, 7, e37762. 30152328 10.7554/eLife.37762 PMC6112853

[ele13335-bib-0128] Shocket, M.S. , Verwillow, A.B. , Numazu, M.G. , Slamani, H. , Cohen, J.M. , Moustaid, F.E. , et al. (2019). Transmission of West Nile virus and other temperate mosquito‐borne viruses occurs at lower environmental temperatures than tropical diseases, bioRxiv, 597898 10.1101/597898

[ele13335-bib-0129] Sinclair, B.J. , Marshall, K.E. , Sewell, M.A. , Levesque, D.L. , Willett, C.S. , Slotsbo, S. , et al. (2016). Can we predict ectotherm responses to climate change using thermal performance curves and body temperatures? Ecol. Lett., 19, 1372–1385. 27667778 10.1111/ele.12686

[ele13335-bib-0130] Siraj, A.S. , Santos‐Vega, M. , Bouma, M.J. , Yadeta, D. , Carrascal, D.R. & Pascual, M. (2014). Altitudinal changes in malaria incidence in highlands of Ethiopia and Colombia. Science, 343, 1154–1158. 24604201 10.1126/science.1244325

[ele13335-bib-0131] Siraj, A.S. , Oidtman, R.J. , Huber, J.H. , Kraemer, M.U.G. , Brady, O.J. , Johansson, M.A. , et al. (2017). Temperature modulates dengue virus epidemic growth rates through its effects on reproduction numbers and generation intervals. PLoS Negl. Trop. Dis., 11, e0005797. 28723920 10.1371/journal.pntd.0005797 PMC5536440

[ele13335-bib-0132] Sloyer, K.E. , Burkett‐Cadena, N.D. , Yang, A. , Corn, J.L. , Vigil, S.L. , McGregor, B.L. , et al. (2019). Ecological niche modeling the potential geographic distribution of four Culicoides species of veterinary significance in Florida, USA. PLoS ONE, 14, e0206648. 30768605 10.1371/journal.pone.0206648 PMC6377124

[ele13335-bib-0133] Smith, D.L. , Guerra, C.A. , Snow, R.W. & Hay, S.I. (2007a). Standardizing estimates of the Plasmodium falciparum parasite rate. Malar. J., 6, 131. 17894879 10.1186/1475-2875-6-131 PMC2072953

[ele13335-bib-0134] Smith, D.L. , McKenzie, F.E. , Snow, R.W. & Hay, S.I. (2007b). Revisiting the basic reproductive number for malaria and its implications for malaria control. PLoS Biol., 5, e42. 17311470 10.1371/journal.pbio.0050042 PMC1802755

[ele13335-bib-0135] Smith, D.L. , Battle, K.E. , Hay, S.I. , Barker, C.M. , Scott, T.W. & McKenzie, F.E. (2012). Ross, macdonald, and a theory for the dynamics and control of mosquito‐transmitted pathogens. PLoS Pathog., 8, e1002588. 22496640 10.1371/journal.ppat.1002588 PMC3320609

[ele13335-bib-0136] Stanaway, J.D. , Shepard, D.S. , Undurraga, E.A. , Halasa, Y.A. , Coffeng, L.E. , Brady, O.J. , et al. (2016). The global burden of dengue: an analysis from the Global Burden of Disease Study 2013. Lancet Infect. Dis., 16, 712–723. 26874619 10.1016/S1473-3099(16)00026-8 PMC5012511

[ele13335-bib-0137] Sternberg, E.D. & Thomas, M.B. (2014). Local adaptation to temperature and the implications for vector‐borne diseases. Trends Parasitol., 30, 115–122. 24513566 10.1016/j.pt.2013.12.010

[ele13335-bib-0138] Stewart Ibarra, A.M. , Ryan, S.J. , Beltrán, E. , Mejía, R. , Silva, M. & Muñoz, Á. (2013). Dengue vector dynamics (Aedes aegypti) influenced by climate and social factors in Ecuador: implications for targeted control. PLoS ONE, 8, e78263. 24324542 10.1371/journal.pone.0078263 PMC3855798

[ele13335-bib-0139] Stewart‐Ibarra, A.M. & Lowe, R. (2013). Climate and non‐climate drivers of dengue epidemics in southern coastal Ecuador. Am. J. Trop. Med. Hyg., 88, 971–981. 23478584 10.4269/ajtmh.12-0478 PMC3752767

[ele13335-bib-0140] Taylor, R.A. , Mordecai, E.A. , Gilligan, C.A. , Rohr, J.R. & Johnson, L.R. (2016). Mathematical models are a powerful method to understand and control the spread of Huanglongbing. PeerJ, 4, e2642. 27833809 10.7717/peerj.2642 PMC5101597

[ele13335-bib-0141] Taylor, R.A. , Ryan, S.J. , Lippi, C.A. , Hall, D.G. , Narouei‐Khandan, H.A. , Rohr, J.R. , et al. (2018). Predicting the fundamental thermal niche of crop pests and diseases in a changing world: a case study on citrus greening, bioRxiv, 465898 10.1101/465898 PMC7367095 32684639

[ele13335-bib-0142] Tesla, B. , Demakovsky, L.R. , Mordecai, E.A. , Ryan, S.J. , Bonds, M.H. , Ngonghala, C.N. , et al. (2018). Temperature drives Zika virus transmission: evidence from empirical and mathematical models. Proc R Soc B, 285, 20180795. 10.1098/rspb.2018.0795 PMC6111177 30111605

[ele13335-bib-0143] Thomas, M.B. & Blanford, S. (2003). Thermal biology in insect‐parasite interactions. Trends Ecol. Evol., 18, 344–350.

[ele13335-bib-0144] Thomas, S.M. , Obermayr, U. , Fischer, D. , Kreyling, J. & Beierkuhnlein, C. (2012). Low‐temperature threshold for egg survival of a post‐diapause and non‐diapause European aedine strain, Aedes albopictus (Diptera: Culicidae). Parasit. Vectors, 5, 100. 22621367 10.1186/1756-3305-5-100 PMC3403971

[ele13335-bib-0145] Thomson, M.C. , Doblas‐Reyes, F.J. , Mason, S.J. , Hagedorn, R. , Connor, S.J. , Phindela, T. , et al. (2006). Malaria early warnings based on seasonal climate forecasts from multi‐model ensembles. Nature, 439, 576–579. 16452977 10.1038/nature04503

[ele13335-bib-0146] Thomson, M.C. , Connor, S.J. , Zebiak, S.E. , Jancloes, M. & Mihretie, A. (2011). Africa needs climate data to fight disease. Nature, 471, 440–442. 21430752 10.1038/471440a

[ele13335-bib-0147] Thomson, M.C. , Ukawuba, I. , Hershey, C.L. , Bennett, A. , Ceccato, P. , Lyon, B. , et al. (2017). Using rainfall and temperature data in the evaluation of national malaria control programs in Africa. Am. J. Trop. Med. Hyg., 97, 32–45. 28990912 10.4269/ajtmh.16-0696 PMC5619931

[ele13335-bib-0148] Tjaden, N.B. , Caminade, C. , Beierkuhnlein, C. & Thomas, S.M. (2018). Mosquito‐borne diseases: Advances in modelling climate‐change impacts. Trends Parasitol., 34, 227–245. 29229233 10.1016/j.pt.2017.11.006

[ele13335-bib-0149] Tompkins, A.M. & Ermert, V. (2013). A regional‐scale, high resolution dynamical malaria model that accounts for population density, climate and surface hydrology. Malar. J., 12, 65. 23419192 10.1186/1475-2875-12-65 PMC3656787

[ele13335-bib-0150] Vogels, C.B.F. , Fros, J.J. , Göertz, G.P. , Pijlman, G.P. & Koenraadt, C.J.M. (2016). Vector competence of northern European Culex pipiens biotypes and hybrids for West Nile virus is differentially affected by temperature. Parasit. Vectors, 9, 393. 27388451 10.1186/s13071-016-1677-0 PMC4937539

[ele13335-bib-0151] Vogels, C.B.F. , Hartemink, N. & Koenraadt, C.J.M. (2017). Modelling West Nile virus transmission risk in Europe: effect of temperature and mosquito biotypes on the basic reproduction number. Sci. Rep., 7, 5022. 28694450 10.1038/s41598-017-05185-4 PMC5504010

[ele13335-bib-0152] Waite, J.L. , Suh, E. , Lynch, P.A. & Thomas, M.B. (2019). Exploring the lower thermal limits for transmission of human malaria, Plasmodium falciparum. Biol. Lett. in press. 10.1098/rsbl.2019.0275 PMC6597502 31238857

[ele13335-bib-0153] Werner, A.K. , Goater, S. , Carver, S. , Robertson, G. , Allen, G.R. & Weinstein, P. (2012). Environmental drivers of ross river virus in southeastern Tasmania, Australia: towards strengthening public health interventions. Epidemiol. Infect., 140, 359–371. 21439102 10.1017/S0950268811000446

[ele13335-bib-0154] Wesolowski, A. , Qureshi, T. , Boni, M.F. , Sundsøy, P.R. , Johansson, M.A. , Rasheed, S.B. , et al. (2015). Impact of human mobility on the emergence of dengue epidemics in Pakistan. Proc. Natl Acad Sci., 112, 11887–11892. 26351662 10.1073/pnas.1504964112 PMC4586847

[ele13335-bib-0155] Wesolowski, A. , Erbach‐Schoenberg, E. , zu, Tatem, A.J., Lourenço, C., Viboud, C., Charu, V.,, , et al. (2017). Multinational patterns of seasonal asymmetry in human movement influence infectious disease dynamics. Nat. Commun., 8, 2069. 29234011 10.1038/s41467-017-02064-4 PMC5727034

[ele13335-bib-0156] Williams, J.W. & Jackson, S.T. (2007). Novel climates, no‐analog communities, and ecological surprises. Front. Ecol. Environ., 5, 475–482.

[ele13335-bib-0157] Yamana, T.K. , Kandula, S. & Shaman, J. (2016). Superensemble forecasts of dengue outbreaks. J. R. Soc. Interface, 13, 20160410. 27733698 10.1098/rsif.2016.0410 PMC5095208

[ele13335-bib-0158] Zouache, K. , Fontaine, A. , Vega‐Rua, A. , Mousson, L. , Thiberge, J.‐M. , Lourenco‐De‐Oliveira, R. , et al. (2014). Three‐way interactions between mosquito population, viral strain and temperature underlying chikungunya virus transmission potential. Proc. R. Soc. B Biol. Sci., 281, 20141078. 10.1098/rspb.2014.1078 PMC4150320 25122228

