# Supplementary material for: Thermal biology of mosquito‐borne disease
Source: Ecol Lett. 2019 Jul 8;22(10):1690–708. doi: 10.1111/ele.13335 (PMC6744319; doi:10.1111/ele.13335)
Supplement: Supplementary file 1 — ‐ [file ELE-22-1690-s001.docx]

**Supplementary Materials for “Thermal biology of mosquito-borne disease”**

Erin A. Mordecai, Jamie M. Caldwell, Marissa Grossman, Catherine A. Lippi, Leah R. Johnson, Marco Neira, Jason R. Rohr, Sadie J. Ryan, Van Savage, Marta S. Shocket, Rachel Sippy, Anna Stewart Ibarra, Matthew B. Thomas, Oswaldo Villena

**Supplementary Tables (Tables S1-S2)**

**Table S1.** Published species distribution models for vectors and vector-borne diseases.

**Table S2.** Trait thermal response functions fit across systems.

**Supplementary Figures (Figures S1-S4)**

**Fig. S1.** Validation of our malaria temperature-dependent *R_0_* model (Mordecai *et al.* 2013) in comparison with a previous model (Parham & Michael 2010).

**Fig. S2.** Validation of our dengue, chikungunya, and Zika virus temperature-dependent *R_0_* model in *Aedes aegypti* (Mordecai *et al.* 2017).

**Fig. S3.** Validation of our West Nile virus temperature-dependent *R_0_* model (Shocket et al., in prep).

**Fig. S4.** Validation of our Ross River virus temperature-dependent *R_0_* model (Shocket *et al.* 2018).

**Table S1. Trait thermal response functional forms, parameters (including thermal limits), and optima.** Medians and 95% CIs. *T_min_*, *T_max_*, and *T_opt_* in degrees Celcius. F(x) is the functional form of the thermal response: B = Brière (*qT*[*T* – *T_min_*][ *T_max_* - *T*]^1/2^), Q = quadratic (–*qT*[*T* – *T_min_*][*T – T_max_*]), Q* = alternative quadratic (*qT*^2^ - *rT* + *s*), and L = linear (*–mT* + *z*). Traits with functional forms Q* and L are listed separately below.

| ***Trait / System*** | ***F(x)*** | ***q* (CIs)** | ***T_min_* (CIs)** | ***T_max_* (CIs)** | ***T_opt_* (CIs)** |
| --- | --- | --- | --- | --- | --- |
| **Biting rate (*a*)** |  |  |  |  |  |
| *Ae. aegypti* | B | 2.02·10^-4^ (1.26–2.88·10^-4^) | 13.8 (7.7–17.1) | 40.0 (40.0–40.4) | 33.8 (33.0–34.3) |
| *Ae. albopictus* | B | 1.90·10^-4^ (1.33–2.68·10^-4^) | 10.4 (5.4–14.4) | 38.1 (36.8–40.9) | 31.8 (30.8–33.7) |
| *An. pseudopunctipennis* | B | 3.43·10^-4^ (1.28–8.21·10^-4^) | 17.1 (2.6–23.3) | 38.7 (35.6–44.2) | 33.2 (30.4–37.3) |
| *Cx. annulirostris* | B | 1.23·10^-4^ (1.02–1.70·10^-4^) | 3.8 (0.7–8.5) | 39.1 (38.0–40.0) | 31.8 (30.8–32.6) |
| *Cx. pipiens* | B | 1.70·10^-4^ (1.18–2.29·10^-4^) | 9.4 (2.8–13.4) | 39.6 (37.9–40.6) | 32.7 (31.3–33.6) |
| *Cx. quinquefasciatus* | B | 7.28·10^-5^ (5.31–11.8·10^-5^) | 3.1 (0.1–10.9) | 39.3 (38.0–40.8) | 31.9 (30.6–33.3) |
| *Cx. tarsalis* | B | 1.67·10^-4^ (0.87–2.56·10^-4^) | 2.3 (0.1–9.4) | 32.0 (30.6–41.7) | 25.9 (24.8–33.9) |
| *Cs. melanura* | B | 1.87·10^-4^ (1.49–2.31·10^-4^) | 7.8 (5.5–11.4) | 31.8 (31.0–33.4) | 26.4 (25.7–27.9) |
| **Fecundity** |  |  |  |  |  |
| *Ae. aegypti* (*EFD*) | B | 8.16·10^-3^ (4.32–15.3·10^-3^) | 14.7 (7.7–20.4) | 34.4 (34.0–36.2) | 29.6 (28.5–31.1) |
| *Ae. albopictus* (*TFD*) | B | 4.77·10^-2^ (3.43–7.01·10^-2^) | 7.9 (3.4–13.3) | 35.6 (35.1–36.9) | 29.4 (28.7–30.6) |
| *Cx. annulirostris* (*EFD*) | Q | 5.65·10^-3^ (3.90–8.71·10^-3^) | 14.8 (11.9–17.2) | 31.3 (30.3–32.9) | 27.0 (26.2–28.4) |
| *Cx. annulirostris* (*nLR*) | Q | 2.75·10^-3^ (1.99–3.29·10^-3^) | 17.1 (15.6–17.9) | 37.1 (36.4–38.4) | 27.2 (26.6–27.6) |
| *Cx. pipiens* (*EFGC*) | Q | 5.98·10^-1^ (4.31–7.91·10^-1^) | 5.3 (2.6–8.5) | 38.9 (36.2–41.8) | 22.1 (20.1–24.4) |
| *Cx. quinquefasciatus* (*ER*) | Q | 6.36·10^-1^ (4.50–9.05·10^-1^) | 5.0 (1.3–9.8) | 37.7 (34.8–40.7) | 21.4 (18.9–24.4) |
| **Proportion ovipositing (*pO*)** |  |  |  |  |  |
| *Cx. pipiens* | Q | 4.45·10^-3^ (2.54–7.77·10^-3^) | 8.2 (4.6–12.1) | 33.2 (30.1–37.5) | 20.8 (18.6–23.4) |
| *Cx. quinquefasciatus* | B | 6.67·10^-4^ (5.80–7.91·10^-4^) | 1.7 (0.2–4.8) | 31.8 (31.1–32.2) | 24.9 (21.8–26.0) |
| *Cs. melanura* | Q | 6.31·10^-3^ (4.52–7.89·10^-3^) | 8.7 (6.9–10.4) | 33.6 (32.5–35.4) | 20.7 (16.9–22.3) |
| **Mosquito Dev. Rate (*MDR*)** |  |  |  |  |  |
| *Ae. aegypti* | B | 7.83·10^-5^ (5.80–10.0·10^-5^) | 11.6 (6.9–14.7) | 39.1 (39.0–39.7) | 32.7 (32.1–33.3) |
| *Ae. albopictus* | B | 6.33·10^-5^ (4.74–8.37·10^-5^) | 8.7 (4.3–12.2) | 39.6 (37.9–41.9) | 32.6 (31.4–34.4) |
| *Ae. camptorhynchus* | B | 4.29·10^-5^ (2.50–8.29·10^-5^) | 9.3 (1.8–19.1) | 38.8 (37.2–40.3) | 32.2 (30.6–34.0) |
| *Ae. notoscriptus* | B | 6.81·10^-5^ (5.43–8.56·10^-5^) | 9.6 (6.4–12.9) | 38.8 (37.1–40.0) | 32.2 (30.8–33.4) |
| *Ae. triseriatus* | B | 4.30·10^-5^ (3.01–5.83·10^-5^) | 0.8 (0–7.5) | 36.5 (34.6–39.5) | 29.3 (27.8–31.9) |
| *Ae. vexans* | B | 4.33·10^-5^ (3.34–5.50·10^-5^) | 1.9 (0.1–10.5) | 38.2 (37.0–39.5) | 30.9 (29.8–32.2) |
| *An. gambiae* | B | 9.52·10^-5^ (4.41–19.6·10^-5^) | 15.4 (10.0–19.9) | 34.7 (33.0–38.3) | 29.9 (28.4–32.7) |
| *Cx. annulirostris* | B | 6.82·10^-5^ (5.47–8.47·10^-5^) | 11.1 (7.7–14.4) | 39.2 (37.9–40.1) | 32.8 (31.6–33.6) |
| *Cx. pipiens* | B | 3.76·10^-5^ (3.36–4.47·10^-5^) | 0.1 (0–4.0) | 38.5 (37.6–39.8) | 30.9 (30.2–31.9) |
| *Cx. quinquefasciatus* | B | 4.14·10^-5^ (3.46–5.26·10^-5^) | 0.1 (0–5.5) | 38.6 (37.4–40.6) | 31.0 (30.0–32.6) |
| *Cx. tarsalis* | B | 4.12·10^-5^ (3.15–5.47·10^-5^) | 4.3 (0–8.4) | 39.9 (37.9–42.2) | 32.3 (31.0–34.0) |
| *Cs. melanura* | B | 2.74·10^-5^ (1.64–4.72·10^-5^) | 8.6 (0–16.8) | 37.6 (35.1–40.4) | 31.1 (28.7–33.7) |
| **Immature survival** |  |  |  |  |  |
| *Ae. aegypti* (*p_EA_*) | Q | 5.99·10^-3^ (5.16–6.85·10^-3^) | 13.6 (12.5–14.5) | 38.3 (37.6–39.0) | 25.9 (25.4–26.4) |
| *Ae. albopictus* (*p_EA_*) | Q | 3.56·10^-3^ (2.65–4.84·10^-3^) | 9.1 (6.3–11.6) | 39.3 (37.2–41.6) | 24.2 (22.6–25.7) |
| *Ae. camptorhynchus* (*p_LA_*) | Q | 2.88·10^-3^ (1.99–4.83·10^-3^) | 5.3 (1.9–10.1) | 38.6 (35.7–42.3) | 22.0 (20.2–24.6) |
| *Ae. notoscriptus* (*p_LA_*) | Q | 5.68·10^-3^ (4.37–7.32·10^-3^) | 9.1 (7.1–11.0) | 36.2 (34.7–37.9) | 20.0 (17.0–23.6) |
| *Ae. triseriatus* (*p_LA_*) | Q | 3.26·10^-3^ (1.95–5.18·10^-3^) | 8.3 (4.9–11.4) | 35.7 (32.9–39.7) | 22.0 (19.9–24.6) |
| *Ae. vexans* (*p_LA_*) | Q | 3.29·10^-3^ (2.65–4.24·10^-3^) | 9.1 (8.1–10.6) | 40.8 (38.4–43.6) | 25.0 (23.9–26.2) |
| *An. gambiae* (*p_EA_*) | Q | 7.65·10^-3^ (4.20–12.5·10^-3^) | 14.5 (11.4–16.4) | 34.1 (32.1–36.6) | 24.3 (22.9–25.5) |
| *Cx. annulirostris* (*p_LA_*) | Q | 5.35·10^-3^ (4.31–6.65·10^-3^) | 14.9 (13.3–16.5) | 39.1 (37.9–40.4) | 27.0 (26.0–28.0) |
| *Cx. pipiens* (*p_LA_*) | Q | 3.60·10^-3^ (2.96–4.42·10^-3^) | 7.8 (6.1–9.3) | 38.4 (37.1–39.9) | 23.1 (22.2–24.0) |
| *Cx. quinquefasciatus* (*p_LA_*) | Q | 4.26·10^-3^ (3.51–5.17·10^-3^) | 8.9 (7.6–9.9) | 37.7 (36.2–39.2) | 23.3 (22.5–24.0) |
| *Cx. tarsalis* (*p_LA_*) | Q | 2.12·10^-3^ (1.52–3.08·10^-3^) | 5.9 (3.0–8.8) | 43.1 (39.8–47.5) | 24.6 (22.9–26.4) |
| *Cs. melanura* (*p_LA_*) | Q | 3.03·10^-3^ (1.55–5.68·10^-3^) | 10.1 (5.7–15.1) | 36.2 (32.8–40.7) | 23.2 (20.4–26.5) |
| **Egg viability (*EV*)** |  |  |  |  |  |
| *Cx. annulirostris* | Q | 6.44·10^-3^ (3.37–12.7·10^-3^) | 14.5 (10.6–17.2) | 38.0 (33.7–42.0) | 26.2 (22.6–27.8) |
| *Cx. pipiens* | Q | 2.11·10^-3^ (1.36–3.05·10^-3^) | 3.2 (0.5–7.1) | 42.6 (39.7–48.3) | 23.0 (20.7–26.3) |
| *Cx. quinquefasciatus* | B | 0.47·10^-3^ (0.34–0.62·10^-3^) | 13.6 (9.3–16.8) | 38.0 (37.2–38.7) | 32.1 (31.3–32.7) |
| *Cx. theileri* | Q | 2.54·10^-3^ (1.86–3.41·10^-3^) | 5.5 (2.6–8) | 45.4 (42.4–49.0) | 23.6 (18.2–27.0) |
| **Lifespan (*lf*)** |  |  |  |  |  |
| *Ae. aegypti–DENV* | Q | 1.44·10^-1^ (1.05–2.21·10^-1^) | 9.0 (6.7–12.5) | 37.7 (35.8–40.0) | 23.4 (22.2–24.8) |
| *Ae. aegypti–ZIKV* | Q | 2.96·10^-1^ (1.48–4.89·10^-1^) | 11.7 (5.1–14.5) | 37.2 (34.9–39.7) | 24.4 (21.7–25.8) |
| *Ae. albopictus* | Q | 13.9·10^-1^ (7.73–23.1·10^-1^) | 13.5 (10.4–16) | 31.4 (29.5–34.0) | 22.5 (21.1–23.7) |
| *Cx. annulirostris* | Q | 2.42·10^-1^ (1.77–3.04·10^-1^) | 13.1 (10.6–14.6) | 33.6 (33.0–34.5) | 23.4 (22.4–24.0) |
| **Transmission probability (*b*)** |  |  |  |  |  |
| DENV \| *Ae. aegypti* | B | 8.33·10^-4^ (5.42–12.4·10^-4^) | 17.2 (12.1–20.9) | 35.8 (35.1–36.8) | 31.0 (29.9–32.1) |
| DENV \| *Ae. albopictus* | B | 7.23·10^-4^ (4.62–10.7·10^-4^) | 16.0 (11.3–19.8) | 36.3 (36.0–37.1) | 31.2 (29.9–32.1) |
| ZIKV \| *Ae. aegypti* | B | 3.44·10^-3^ (1.83–5.65·10^-3^) | 22.9 (20.5–23.9) | 38.4 (36.6–40.5) | 30.6 (29.6–31.5) |
| SLEV \| *Cx. tarsalis* | Q | 2.98·10^-3^ (1.63–5.31·10^-3^) | 10.8 (6.2–14.2) | 41.6 (36.8–49.1) | 26.2 (23.5–29.7) |
| WEEV \| *Cx. tarsalis* | Q | 3.17·10^-3^ (1.65–5.06·10^-3^) | 8.2 (5.1–10.7) | 33.5 (31.0–38.9) | 20.9 (19.2–23.2) |
| WNV \| *Cx. tarsalis* | Q | 2.94·10^-3^ (1.91–4.48·10^-3^) | 11.3 (7.6–14.0) | 41.9 (37.7–47.0) | 26.6 (23.9–29.3) |
| **Infection probability (*c*)** |  |  |  |  |  |
| DENV \| *Ae. aegypti* | B | 4.88·10^-4^ (3.43–6.53·10^-4^) | 12.7 (4.3–17.1) | 37.4 (35.8–39.5) | 31.5 (29.6–33.5) |
| DENV \| *Ae. albopictus* | B | 4.33·10^-4^ (3.37–5.78·10^-4^) | 2.8 (0–11.2) | 36.7 (36.1–38.2) | 29.6 (24.7–31.3) |
| SINV \| *Ae. taeniorhynchus* | Q | 1.24·10^-3^ (0.75–2.17·10^-3^) | 1.4 (0–9.1) | 48.4 (40.8–57.1) | 25.4 (21.0–31.1) |
| SINV \| *Cx. pipiens* | Q | 1.33·10^-3^ (0.47–2.30·10^-3^) | 0 (0–0) | 35.0 (28.1–61.1) | 17.5 (14.1–30.5) |
| WNV \| *Cx. pipiens* | Q | 2.56·10^-3^ (2.05–3.19·10^-3^) | 15.6 (14.3–16.6) | 52.2 (48.4–56.6) | 33.9 (31.9–36.1) |
| SLEV \| *Cx. tarsalis* | Q | 2.03·10^-3^ (1.28–3.07·10^-3^) | 8.8 (6.6–10.6) | 43.7 (38.9–51.4) | 26.2 (24.2–29.7) |
| WEEV \| *Cx. tarsalis* | Q | 3.04·10^-3^ (2.52–3.68·10^-3^) | 1.3 (0.4–2.9) | 38.8 (36.7–41.5) | 15.5 (13.4–19.7) |
| **Vector competence (*bc*)** |  |  |  |  |  |
| RVFV \| *Ae. taeniorhynchus* | Q | 1.51·10^-3^ (1.03–2.05·10^-3^) | 7.1 (2.8–9.8) | 42.3 (39.3–46.5) | 24.7 (22.0–27.0) |
| EEEV \| *Ae. triseriatus* | Q | 1.51·10^-3^ (0.96–2.24·10^-3^) | 7.0 (2.9–11.9) | 50.3 (42.3–63.1) | 28.8 (23.6–35.8) |
| *P. fal.*\| *An. quadrimaculatus* | Q | 6.13·10^-3^ (4.85–7.63·10^-3^) | 12.6 (11.2–13.8) | 35.9 (34.4–37.6) | 24.3 (23.3–25.2) |
| RRV \| *Cx. annulirostris* | Q | 2.78·10^-3^ (2.02–4.27·10^-3^) | 6.1 (1.6–9.6) | 42.8 (39.6–44.9) | 24.4 (22.4–25.6) |
| WNV \| *Cx. pipiens* | Q | 3.05·10^-3^ (1.68–4.87·10^-3^) | 16.8 (15–17.9) | 38.9 (36.1–44.1) | 27.8 (26.6–30.1) |
| WEEV \| *Cx. tarsalis* | Q | 1.17·10^-3^ (0.55–2.36·10^-3^) | 5.1 (0.6–13.3) | 37.0 (33.5–46.0) | 21.4 (18.1–27.3) |
| WNV \| *Cx. univittatus* | Q | 2.32·10^-3^ (1.58–3.68·10^-3^) | 4.2 (1.5–7.1) | 45.2 (39.6–53.0) | 23.7 (19.4–27.3) |
| **Parasite Dev. Rate (*PDR*)** |  |  |  |  |  |
| DENV \| *Ae. aegypti* | B | 6.13·10^-5^ (3.98–11.9·10^-5^) | 10.3 (3.7–18.2) | 45.6 (40.1–53.5) | 37.7 (33.8–43.8) |
| ZIKV \| *Ae. aegypti* | B | 1.68·10^-4^ (0.62–3.21·10^-4^) | 19.7 (4.9–23.7) | 42.5 (38.8–44.9) | 36.5 (33.2–38.9) |
| RVFV \| *Ae. taeniorhynchus* | B | 8.84·10^-5^ (2.51–15.5·10^-5^) | 9.0 (5.4–13.8) | 45.9 (41.9–50.3) | 37.8 (34.5–41.3) |
| EEEV \| *Ae. triseriatus* | B | 7.05·10^-5^ (5.21–9.68·10^-5^) | 11.6 (7.0–16.4) | 44.8 (40.6–49.4) | 37.2 (33.8–41.1) |
| *P. fal.*\| *Anopheles* spp. | B | 1.06·10^-4^ (0.88–1.26·10^-4^) | 14.1 (13.1–15.2) | 34.2 (33.5–34.8) | 29.2 (28.7–29.7) |
| MVEV \| *Cx. annulirostris* | B | 1.01·10^-4^ (0.64–1.55·10^-4^) | 12.8 (6.6–19.0) | 41.9 (38.6–45.0) | 35.2 (32.2–37.8) |
| RRV \| *Cx. annulirostris* | B | 1.34·10^-4^ (0.86–2.15·10^-4^) | 5.9 (1.3–13.0) | 41.3 (36.1–45.0) | 33.6 (29.6–37.0) |
| WNV \| *Cx. pipiens* | B | 7.38·10^-5^ (5.38–9.94·10^-5^) | 11.4 (7.3–15.0) | 45.2 (40.7–50.3) | 37.5 (33.8–41.6) |
| WNV \| *Cx. quinquefasciatus* | B | 7.12·10^-5^ (4.58–10.2·10^-5^) | 19.0 (12.9–21.0) | 44.1 (38.8–50.4) | 37.7 (33.6–42.7) |
| SLEV \| *Cx. tarsalis* | B | 7.11·10^-5^ (5.60–8.95·10^-5^) | 12.8 (10.3–14.3) | 45.2 (40.2–51.5) | 37.7 (33.8–42.6) |
| WEEV \| *Cx. tarsalis* | B | 6.43·10^-5^ (4.44–10.4·10^-5^) | 4.0 (0–12.6) | 44 .0(38.3–50.9) | 35.7 (31.0–41.4) |
| WNV \| *Cx. tarsalis* | B | 6.57·10^-5^ (5.11–8.85·10^-5^) | 11.2 (7.9–14.9) | 44.7 (40.4–49.4) | 37.0 (33.6–40.9) |
| WNV \| *Cx. univittatus* | B | 7.54·10^-5^ (4.13–11.1·10^-5^) | 10.2 (7.1–15.3) | 34.4 (31.2–51.1) | 28.8 (26.1–42.5) |

| ***Trait / System*** | ***F(x)*** | ***q* (CIs)** | ***r* (CIs)** | ***s* (CIs)** |
| --- | --- | --- | --- | --- |
| **Mortality rate (**$\boldsymbol{\mu}$**)** |  |  |  |  |
| *An. gambiae* | Q* | 1.10·10^-3^ (9.29·10^-4^ – 1.27·10^-3^) | 4.90·10^-2^ (4.10·10^-2^ – 5.66·10^-2^) | 0.609 (0.527 – 0.682) |

| ***Trait / System*** | ***F(x)*** | **m (CIs)** | ***z* (CIs)** | ***Tmax = z/m* (CIs)** |
| --- | --- | --- | --- | --- |
| **Lifespan (*lf*)** |  |  |  |  |
| *Ae. taeniorhynchus* | L | 2.02 (1.59–3.19) | 85.9 (73.8–117.6) | 42.7 (34.5–48.5) |
| *Cx. pipiens* | L | 4.86 (3.83–5.84) | 169.8 (142.1–195.6) | 34.9 (32.9–37.9) |
| *Cx. quinquefasciatus* | L | 3.80 (1.85–5.29) | 136.3 (86.8–174.0) | 35.9 (32.1–48.5) |
| *Cx. tarsalis* | L | 1.69 (1.12–2.24) | 69.6 (55.8–83.5) | 41.3 (36.6–50.8) |

**Table S2. Published species distribution models for vectors and vector-borne diseases.** Columns indicate the study, pathogen species (where applicable), vector species, vector life stage (where applicable), the temperature measurement(s) included in the model, and the reported relationship between temperature and occurrence probability.

| **Reference** | **Disease** | **Vector/Host** | **Life stage** | **Model** | **Temperature measures** | **Relationship reported** |
| --- | --- | --- | --- | --- | --- | --- |
| (Bishop *et al.* 2000) | Bluetongue | *Culicoides brevitarsis* | Adult | LOESS | Weekly or monthly mean temperature | Time from occurrence and distance from site |
| (Calvete *et al.* 2009) | Bluetongue | Animal | NA | GLM | Annual mean temperature | Parameters |
| (Cappai *et al.* 2018) | Bluetongue | Sheep, goat | NA | Logistic mixed model | Decadal mean temperature | Odds ratios |
| (Conte *et al.* 2004) | Bluetongue | *Cu. imicola* | Adult | Spatial process model | Daily minimum temperature | Not given |
| (Gao *et al.* 2017) | Bluetongue | Sheep | NA | MaxEnt | Monthly mean temperature, monthly max temperature, monthly min temperature, monthly temperature range | Not given |
| (Mukhopadhyay *et al.* 2017) | Bluetongue | *Cu. orientalis* | Adult | MaxEnt | Bioclim (annual mean temperature, mean temperature of coldest quarter, mean temperature of warmest quarterly) | Not given |
| (Purse *et al.* 2004) | Bluetongue | *Cu. pulicaris, Cu. newsteadi, Cu. obsoletus, Cu. imicola* | Adult | Stepwise discriminant analysis | Mean, variance, and minimum land surface temperature, minimum air temperature | Not given |
| (Rigot *et al.* 2012) | Bluetongue | *Cu. imicola* | Adult | GLM | Daily minimum and maximum temp, monthly mean, minimum, and maximum land surface temperature | Parameters |
| (Tatem *et al.* 2003) | Bluetongue | *Cu. imicola* | Adult | Stepwise discriminant analysis | Monthly maximum land surface temperature, monthly mean air temperature, monthly variance land surface temperature | Not given |
| (Escobar *et al.* 2016a) | CHIKV | *Aedes aegypti, Aedes albopictus* | NA | NicheA | WorldClim (annual mean temperature, mean diurnal temperature range, isothermality, temperature seasonality, maximum temperature of the warmest month, minimum temperature of the coldest month, temperature annual range, mean temperature of warmest quarter, mean temperature of coldest quarter) | Central values of climate conditions studied were around 25°C, the temperature corresponding to high reported CHIKV R_o_ |
| (Nsoesie *et al.* 2016) | CHIKV | *Aedes aegypti, Aedes albopictus* | NA | Boosted regression trees | Land surface temperature | Not given |
| (Bhatt *et al.* 2013) | DENV | *Aedes aegypti* | NA | Boosted regression trees | Monthly minimum, maximum, and mean temperature restricted by DENV transmission suitability | Not given |
| (Mweya *et al.* 2016) | DENV | *Aedes aegypti* | Larvae, pupae, adults | MaxEnt | Bioclim (annual temperature range, mean temperature of warmest quarter, temperature seasonality, minimum temperature of coldest month, maximum temperature of warmest month, mean diurnal range) | Not given |
| (Alegana *et al.* 2014) | Malaria | Human | NA | Bayesian conditional autoregressive model | Temperature suitability index | Parameters |
| (Foley *et al.* 2014) | Malaria | *Anopheles albitarsis, An. deaneorum, An. janconnae, An. marajoara, An. oryzalimnetes, An. albitarsis F, An. albitarsis G, An. albitarsis I, An. albitarsis* | Adult | MaxEnt | Bioclim (mean diurnal range, minimum temperature of coldest month, annual temperature range, mean temperature of wettest quarter, mean temperature of driest quarter, mean temperature of warmest quarter, mean temperature of coldest quarter) | Not given |
| (Hundessa *et al.* 2018) | Malaria | Human | NA | GAM | Annual mean temperature | Effect size |
| (Arsevska *et al.* 2016) | RVF | *Aedes vexans, Anopheles multicolor, Culex perexiguus,* | NA | Multiple criteria decision making with weighted linear combination | Monthly mean temperature | Description and range of function |
| (Clements *et al.* 2006) | RVF | Human, animal | NA | Multiple criteria decision making with weighted linear combination | Land surface temperature | Description and range of function |
| (Clements *et al.* 2007) | RVF | Cattle, goat, sheep | NA | Bayesian spatial model | Mean land surface temperature | Coefficients and odds ratios |
| (Conley *et al.* 2014) | RVF | *Cx. pipiens* | Larvae, adult | MaxEnt | Bioclim (mean diurnal range, maximum temperature of warmest month, minimum temperature of coldest month, annual temperature range, mean temperature of wettest/direst/warmest quarter) | Not given |
| (Conley *et al.* 2014) | RVF | *Cx. pipiens* | Larvae, adult | Boosted regression trees | Bioclim (mean diurnal range, maximum temperature of warmest month, minimum temperature of coldest month, annual temperature range, mean temperature of wettest/direst/warmest quarter) | Not given |
| (Mweya *et al.* 2013) | RVF | *Cx. pipiens* | Adult | MaxEnt | Bioclim (isothermality, annual temperature range, mean temperature of warmest quarter, mean temperature of coolest quarter) | Not given |
| (Mweya *et al.* 2017) | RVF | *Cx. pipiens* | Adult | MaxEnt | Bioclim (mean temperature of warmest quarter, isothermality) | Not given |
| (Sallam *et al.* 2013) | RVF | *Cx. tritaeniorhynchus* | Larvae | MaxEnt | Bioclim (mean temperature of wettest quarter, mean temperature of driest quarter, mean diurnal range) | Not given |
| (Sánchez-Vizcaíno *et al.* 2013) | RVF | Human | NA | Multiple criteria decision making with weighted linear combination | Monthly mean temperature | Suitability and temperature |
| (Gleiser & Gorla 2007) | WEE | *Oc. albifasciatus* | Adult | Discriminant function analysis | Land surface temperature | Standardized coefficients |
| (Conley *et al.* 2014) | WNV | *Cx. pipiens* | Larvae, adult | MaxEnt | Bioclim (mean diurnal range, maximum temperature of warmest month, minimum temperature of coldest month, annual temperature range, mean temperature of wettest/direst/warmest quarter) | Not given |
| (Conley *et al.* 2014) | WNV | *Cx. pipiens* | Larvae, adult | Boosted regression trees | Bioclim (mean diurnal range, maximum temperature of warmest month, minimum temperature of coldest month, annual temperature range, mean temperature of wettest/direst/warmest quarter) | Not given |
| (Mongoh *et al.* 2007) | WNV | Horse | NA | Principle component analysis | Mean temperature | Estimates |
| (Tran *et al.* 2014) | WNV | Human | NA | Logistic regression model | Temperature anomalies | Parameters |
| (Ward 2005) | WNV | Horse | NA | Probability distribution model | Daily maximum temperature | Cumulative probabilities |
| (Ward *et al.* 2009) | WNV | Horse | NA | Bayesian spatial lag model | Mean temperature | Coefficient |
| (Messina *et al.* 2016) | ZIKV | *Aedes aegypti, Aedes albopictus* | NA | Boosted regression trees | WorldClim restricted by DENV transmission suitability | Not given |
| (Samy *et al.* 2016) | ZIKV | *Aedes aegypti, Aedes albopictus* | NA | MaxEnt | Land surface temperature | Not given |
| (Beebe *et al.* 2009) |  | *Aedes aegypti* | NA | GARP | Annual mean temperature, mean maximum and minimum temperatures for months of Jan and July | Not given |
| (Benedict *et al.* 2007) |  | *Aedes albopictus* | NA | GARP | Annual mean temperature, annual mean maximum temperature, annual mean minimum temperature, average DTR | Annual values for climatic environmental variables reported for georeferenced points |
| (Bogoch *et al.* 2016) |  | *Aedes aegypti, Aedes albopictus* | Eggs, larvae, pupae, adults | Boosted regression trees | Masked temperature suitability determined by dynamic population-level simulation | Not given |
| (Campbell *et al.* 2015) |  | *Aedes aegypti, Aedes albopictus* | NA | MaxEnt | WorldClim (monthly average maximum temperature, monthly average minimum temperature) | Not given |
| (Cardoso-Leite *et al.* 2014) |  | *Aedes aegypti* | NA | MaxEnt | Bioclim (annual mean temperature, mean diurnal range, isothermality, temperature seasonality, maximum temperature of warmest month, minimum temperature of coldest month, temperature annual range, mean temperature of wettest quarter, mean temperature of driest quarter, mean temperature of warmest quarter, mean temperature of coldest quarter) | Not given |
| (Cunze *et al.* 2016) |  | *Aedes albopictus, Aedes japonicus* | NA | MaxEnt and ensemble forecasting | WorldClim (mean temperature of coldest quarter, mean temperature of warmest quarter, temperature annual range) | Not given |
| (Escobar *et al.* 2016b) |  | *Aedes aegypti, Aedes albopictus* | NA | MaxEnt | Land surface temperature | Not given |
| (Koch *et al.* 2016) |  | *Aedes albopictus* | NA | MaxEnt | WorldClim (annual mean temperature, mean temperature of warmest quarter, mean temperature of coldest quarter) | One variable response curves of modeled habitat suitability and temperate measures |
| (Kraemer *et al.* 2015) |  | *Aedes aegypti, Aedes albopictus* | Eggs, larvae, pupae, adults | Boosted regression trees | Temperature suitability based on previously published index | Not given |
| (Ogden *et al.* 2014) |  | *Aedes albopictus* | NA | Scaled suitability and sigmoidal function | Climatological indicators (thresholds for annual temperature, January temperature, summer temperature) | Not given |
| (Rochlin *et al.* 2013) |  | *Aedes albopictus* | Adult | MaxEnt | WorldClim (mean temperature of coldest quarter) | Null presence probability below -2°C mean winter temperature |
| (Shabani *et al.* 2018) |  | *Aedes albopictus* | Eggs, larvae, pupae, adults | MaxEnt | Bioclim (annual mean temperature of coldest quarter) | Highest probabilities of presence in areas where coldest mean quarter temperature ranged from 16-23°C |

**
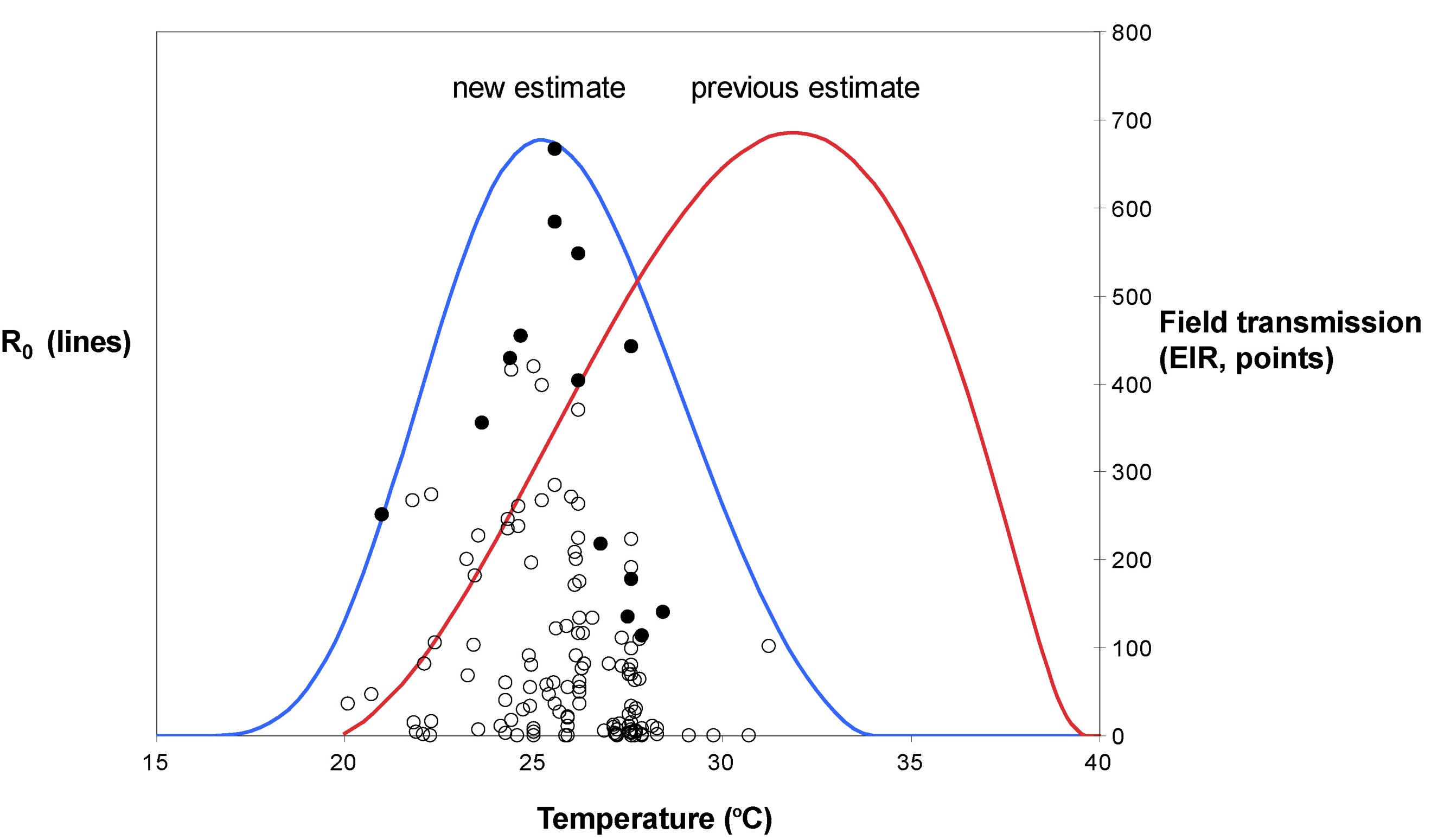
**

**Figure S1.** Validation of our malaria temperature-dependent *R_0_* model (blue line; Mordecai *et al.* 2013) in comparison with a previous model (red line; Parham & Michael 2010). Data points are independent field data on entomological inoculation rate (EIR) across Africa spanning 30 years (Hay *et al.* 2000) plotted against average temperature during the transmission season. Filled circles are the maximum EIR within each group of data points, binned by temperature (where we expect malaria to be the least controlled by other factors and therefore most sensitive to temperature). Open circles are the remaining data points. Reproduced with permission from (Mordecai *et al.* 2013)

**
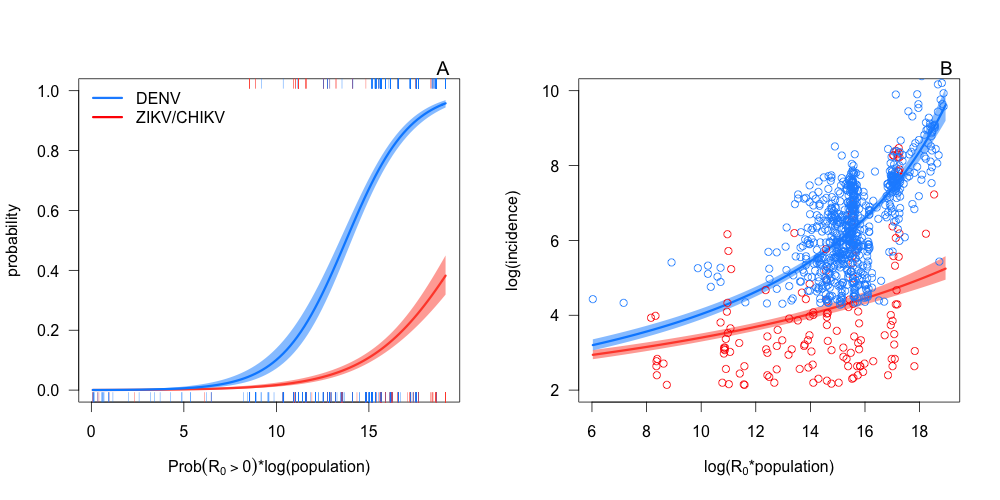
**

**Fig. S2.** Validation of our dengue, chikungunya, and Zika virus temperature-dependent *R_0_* model in *Aedes aegypti* (Mordecai *et al.* 2017). Lines compare the model-predicted dengue (blue) and chikungunya and Zika (red) probability occurrence of local transmission (A) and natural log of the incidence given that local transmission occurred (B) with weekly data country-scale data from the Americas in 2014-2016, plotted against the mean temperature 10 weeks prior (hashes in A and points in B). This 10-week lag represents our *a priori* expectation of the time that elapses between suitable temperatures and reported human cases (Stewart Ibarra *et al.* 2013; Mordecai *et al.* 2017). Predicted lines and 95% credible intervals (shaded areas) are the results of generalized linear models where the predictors are the posterior probability that *R_0_*(*T*) > 0 times the log of population size (A), and the log of *R_0_*(*T*) times the population size (B). These metrics reflect our *a priori* expectation of the interactive relationship between *R_0_* and population size in driving epidemic occurrence and incidence. Reproduced with permission from (Mordecai *et al.* 2017).

**Fig. S3.** Validation of our West Nile virus temperature-dependent *R_0_* model (Shocket et al., in prep). Points are incidence of human neuroinvasive West Nile disease across US counties, plotted against mean temperature from May – September from 2001-2016 for bins of 42 counties (+/- standard errors; total N = 3,109). Line is a LOESS regression illustrating the unimodal relationship between temperature and disease incidence with a peak at 24°C. Reproduced with permission from (Shocket et al., in prep).

**Fig. S4.** Validation of our Ross River virus temperature-dependent *R_0_* model (Shocket *et al.* 2018). Average monthly human Ross River virus cases in Australia from 1992-2013 (gray bars) and average *R_0_* (line) plotted against average monthly temperature. Averages for temperature and *R_0_* are calculated across the 15 largest cities in Australia, weighted by population size. *R_0_* predicts the seasonality of Ross River virus incidence with a 2-month lag, matching our a priori expectation of the time that elapses between suitable temperatures and reported human cases (Stewart Ibarra *et al.* 2013; Mordecai *et al.* 2017). Reproduced with permission from (Shocket *et al.* 2018).

**References Cited in Supplementary Materials**

Alegana, V.A., Wright, J.A., Nahzat, S.M., Butt, W., Sediqi, A.W., Habib, N., *et al.* (2014). Modelling the Incidence of Plasmodium vivax and Plasmodium falciparum Malaria in Afghanistan 2006–2009. *PLoS ONE*, 9, e102304.

Arsevska, E., Hellal, J., Mejri, S., Hammami, S., Marianneau, P., Calavas, D., *et al.* (2016). Identifying Areas Suitable for the Occurrence of Rift Valley Fever in North Africa: Implications for Surveillance. *Transbound. Emerg. Dis.*, 63, 658–674.

Beebe, N.W., Cooper, R.D., Mottram, P. & Sweeney, A.W. (2009). Australia’s Dengue Risk Driven by Human Adaptation to Climate Change. *PLoS Negl. Trop. Dis.*, 3, e429.

Benedict, M.Q., Levine, R.S., Hawley, W.A. & Lounibos, L.P. (2007). Spread of The Tiger: Global Risk of Invasion by The Mosquito Aedes albopictus. *Vector-Borne Zoonotic Dis.*, 7, 76–85.

Bhatt, S., Gething, P.W., Brady, O.J., Messina, J.P., Farlow, A.W., Moyes, C.L., *et al.* (2013). The global distribution and burden of dengue. *Nature*, 496, 504–507.

Bishop, A.L., Barchia, I.M. & Spohr, L.J. (2000). Models for the dispersal in Australia of the arbovirus vector, Culicoides brevitarsis Kieffer (Diptera: Ceratopogonidae). *Prev. Vet. Med.*, 47, 243–254.

Bogoch, I.I., Brady, O.J., Kraemer, M.U.G., German, M., Creatore, M.I., Kulkarni, M.A., *et al.* (2016). Anticipating the international spread of Zika virus from Brazil. *The Lancet*, 387, 335–336.

Calvete, C., Estrada, R., Miranda, M.A., Borrás, D., Calvo, J.H. & Lucientes, J. (2009). Ecological correlates of bluetongue virus in Spain: Predicted spatial occurrence and its relationship with the observed abundance of the potential Culicoides spp. vector. *Vet. J.*, 182, 235–243.

Campbell, L.P., Luther, C., Moo-Llanes, D., Ramsey, J.M., Danis-Lozano, R. & Peterson, A.T. (2015). Climate change influences on global distributions of dengue and chikungunya virus vectors. *Philos. Trans. R. Soc. B Biol. Sci.*, 370, 20140135.

Cappai, S., Loi, F., Coccollone, A., Contu, M., Capece, P., Fiori, M., *et al.* (2018). Retrospective analysis of Bluetongue farm risk profile definition, based on biology, farm management practices and climatic data. *Prev. Vet. Med.*, 155, 75–85.

Cardoso-Leite, R., Vilarinho, A.C., Novaes, M.C., Tonetto, A.F., Vilardi, G.C. & Guillermo-Ferreira, R. (2014). Recent and future environmental suitability to dengue fever in Brazil using species distribution model. *Trans. R. Soc. Trop. Med. Hyg.*, 108, 99–104.

Clements, A.C., Pfeiffer, D.U. & Martin, V. (2006). Application of knowledge-driven spatial modelling approaches and uncertainty management to a study of Rift Valley fever in Africa. *Int. J. Health Geogr.*, 5, 57.

Clements, A.C.A., Pfeiffer, D.U., Martin, V., Pittiglio, C., Best, N. & Thiongane, Y. (2007). Spatial Risk Assessment of Rift Valley Fever in Senegal. *Vector-Borne Zoonotic Dis.*, 7, 203–216.

Conley, A.K., Fuller, D.O., Haddad, N., Hassan, A.N., Gad, A.M. & Beier, J.C. (2014). Modeling the distribution of the West Nile and Rift Valley Fever vector Culex pipiens in arid and semi-arid regions of the Middle East and North Africa. *Parasit. Vectors*, 7, 289.

Conte, A., Ippoliti, C., Calistri, P., Pelini, S., Savini, L., Salini, R., *et al.* (2004). Towards the identification of potential infectious sites for bluetongue in Italy: a spatial analysis approach based on the distribution of Culicoides imicola. *Vet. Ital.*, 40, 311–315.

Cunze, S., Koch, L.K., Kochmann, J. & Klimpel, S. (2016). Aedes albopictus and Aedes japonicus - two invasive mosquito species with different temperature niches in Europe. *Parasit. Vectors*, 9, 573.

Escobar, L.E., Qiao, H. & Peterson, A.T. (2016a). Forecasting Chikungunya spread in the Americas via data-driven empirical approaches. *Parasit. Vectors*, 9, 112.

Escobar, L.E., Romero-Alvarez, D., Leon, R., Lepe-Lopez, M.A., Craft, M.E., Borbor-Cordova, M.J., *et al.* (2016b). Declining Prevalence of Disease Vectors Under Climate Change. *Sci. Rep.*, 6, 39150.

Foley, D.H., Linton, Y.-M., Ruiz‐Lopez, J.F., Conn, J.E., Sallum, M.A.M., Póvoa, M.M., *et al.* (2014). Geographic distribution, evolution, and disease importance of species within the Neotropical Anopheles albitarsis Group (Diptera, Culicidae). *J. Vector Ecol.*, 39, 168–181.

Gao, X., Qin, H., Xiao, J. & Wang, H. (2017). Meteorological conditions and land cover as predictors for the prevalence of Bluetongue virus in the Inner Mongolia Autonomous Region of Mainland China. *Prev. Vet. Med.*, 138, 88–93.

Gleiser, R.M. & Gorla, D.E. (2007). Predicting the spatial distribution of Ochlerotatus albifasciatus (Diptera: Culicidae) abundance with NOAA imagery. *Bull. Entomol. Res.*, 97, 607–612.

Hay, S.I., Rogers, D.J., Toomer, J.F. & Snow, R.W. (2000). Annual Plasmodium falciparum entomological inoculation rates (EIR) across Africa: literature survey, internet access and review. *Trans. R. Soc. Trop. Med. Hyg.*, 94, 113–127.

Hundessa, S., Williams, G., Li, S., Liu, D.L., Cao, W., Ren, H., *et al.* (2018). Projecting potential spatial and temporal changes in the distribution of Plasmodium vivax and Plasmodium falciparum malaria in China with climate change. *Sci. Total Environ.*, 627, 1285–1293.

Koch, L.K., Cunze, S., Werblow, A., Kochmann, J., Dörge, D.D., Mehlhorn, H., *et al.* (2016). Modeling the habitat suitability for the arbovirus vector Aedes albopictus (Diptera: Culicidae) in Germany. *Parasitol. Res.*, 115, 957–964.

Kraemer, M.U., Sinka, M.E., Duda, K.A., Mylne, A.Q., Shearer, F.M., Barker, C.M., *et al.* (2015). The global distribution of the arbovirus vectors Aedes aegypti and Ae. albopictus. *eLife*, 4, e08347.

Messina, J.P., Kraemer, M.U., Brady, O.J., Pigott, D.M., Shearer, F.M., Weiss, D.J., *et al.* (2016). Mapping global environmental suitability for Zika virus. *eLife*, 5.

Mongoh, M.N., Khaitsa, M.L. & Dyer, N.W. (2007). Environmental and ecological determinants of West Nile virus occurrence in horses in North Dakota, 2002. *Epidemiol. Infect.*, 135, 57.

Mordecai, E.A., Cohen, J.M., Evans, M.V., Gudapati, P., Johnson, L.R., Lippi, C.A., *et al.* (2017). Detecting the impact of temperature on transmission of Zika, dengue, and chikungunya using mechanistic models. *PLoS Negl. Trop. Dis.*, 11, e0005568.

Mordecai, E.A., Paaijmans, K.P., Johnson, L.R., Balzer, C.H., Ben-Horin, T., de Moor, E., *et al.* (2013). Optimal temperature for malaria transmission is dramatically lower than previously predicted. *Ecol. Lett.*, 16, 22–30.

Mukhopadhyay, E., Hazra, S., Saha, G.K. & Banerjee, D. (2017). Altitudinal variation and bio-climatic variables influencing the potential distribution of Culicoides orientalis Macfie, 1932, suspected vector of Bluetongue virus across the North Eastern Himalayan belt of Sikkim. *Acta Trop.*, 176, 402–411.

Mweya, C.N., Kimera, S.I., Kija, J.B. & Mboera, L.E.G. (2013). Predicting distribution of Aedes aegypti and Culex pipiens complex, potential vectors of Rift Valley fever virus in relation to disease epidemics in East Africa. *Infect. Ecol. Epidemiol.*, 3, 21748.

Mweya, C.N., Kimera, S.I., Stanley, G., Misinzo, G. & Mboera, L.E.G. (2016). Climate Change Influences Potential Distribution of Infected Aedes aegypti Co-Occurrence with Dengue Epidemics Risk Areas in Tanzania. *PLOS ONE*, 11, e0162649.

Mweya, C.N., Mboera, L.E.G. & Kimera, S.I. (2017). Climate Influence on Emerging Risk Areas for Rift Valley Fever Epidemics in Tanzania. *Am. J. Trop. Med. Hyg.*, 97, 109–114.

Nsoesie, E., Kraemer, M., Golding, N., Pigott, D., Brady, O., Moyes, C., *et al.* (2016). Global distribution and environmental suitability for chikungunya virus, 1952 to 2015. *Euro Surveill. Bull. Eur. Sur Mal. Transm. Eur. Commun. Dis. Bull.*, 21.

Ogden, N.H., Milka, R., Caminade, C. & Gachon, P. (2014). Recent and projected future climatic suitability of North America for the Asian tiger mosquito Aedes albopictus. *Parasit. Vectors*, 7, 532.

Parham, P. & Michael, E. (2010). Modeling the Effects of Weather and Climate Change on Malaria Transmission. *Environ. Health Perspect.*, 118, 620–626.

Purse, B.V., Caracappa, S., Marino, A.M.F., Tatem, A.J., Rogers, D.J., Mellor, P.S., *et al.* (2004). Modelling the distribution of outbreaks and Culicoides vectors in Sicily: towards predictive risk maps for Italy. *Vet. Ital.*, 40, 303–310.

Rigot, T., Conte, A., Goffredo, M., Ducheyne, E., Hendrickx, G. & Gilbert, M. (2012). Predicting the spatio-temporal distribution of Culicoides imicola in Sardinia using a discrete-time population model. *Parasit. Vectors*, 5, 270.

Rochlin, I., Ninivaggi, D.V., Hutchinson, M.L. & Farajollahi, A. (2013). Climate Change and Range Expansion of the Asian Tiger Mosquito (Aedes albopictus) in Northeastern USA: Implications for Public Health Practitioners. *PLoS One San Franc.*, 8, e60874.

Sallam, M.F., Ahmed, A.M.A., Abdel-Dayem, M.S. & Abdullah, M.A.R. (2013). Ecological Niche Modeling and Land Cover Risk Areas for Rift Valley Fever Vector, Culex tritaeniorhynchus Giles in Jazan, Saudi Arabia. *PLOS ONE*, 8, e65786.

Samy, A.M., Thomas, S.M., Wahed, A.A.E., Cohoon, K.P., Peterson, A.T., Samy, A.M., *et al.* (2016). Mapping the global geographic potential of Zika virus spread. *Mem. Inst. Oswaldo Cruz*, 111, 559–560.

Sánchez-Vizcaíno, F., Martínez-López, B. & Sánchez-Vizcaíno, J.M. (2013). Identification of suitable areas for the occurrence of Rift Valley fever outbreaks in Spain using a multiple criteria decision framework. *Vet. Microbiol.*, 165, 71–78.

Shabani, F., Tehrany, M.S., Solhjouy-fard, S. & Kumar, L. (2018). A comparative modeling study on non-climatic and climatic risk assessment on Asian Tiger Mosquito (Aedes albopictus). *PeerJ*, 6, e4474.

Shocket, M.S., Ryan, S.J. & Mordecai, E.A. (2018). Temperature explains broad patterns of Ross River virus transmission. *eLife*, 7, e37762.

Stewart Ibarra, A.M., Ryan, S.J., Beltrán, E., Mejía, R., Silva, M. & Muñoz, Á. (2013). Dengue vector dynamics (Aedes aegypti) influenced by climate and social factors in Ecuador: implications for targeted control. *PLoS ONE*, 8, e78263.

Tatem, A.J., Baylis, M., Mellor, P.S., Purse, B.V., Capela, R., Pena, I., *et al.* (2003). Prediction of bluetongue vector distribution in Europe and north Africa using satellite imagery. *Vet. Microbiol.*, 97, 13–29.

Tran, A., Sudre, B., Paz, S., Rossi, M., Desbrosse, A., Chevalier, V., *et al.* (2014). Environmental predictors of West Nile fever risk in Europe. *Int. J. Health Geogr.*, 13, 26.

Ward, M.P. (2005). Epidemic West Nile virus encephalomyelitis: A temperature-dependent, spatial model of disease dynamics. *Prev. Vet. Med.*, 71, 253–264.

Ward, M.P., Wittich, C.A., Fosgate, G. & Srinivasan, R. (2009). Environmental risk factors for equine West Nile virus disease cases in Texas. *Vet. Res. Commun.*, 33, 461–471.
